# Managing gastroenteritis in puppies: veterinary practices in Trinidad and Tobago and Barbados

**DOI:** 10.3389/fvets.2026.1813429

**Published:** 2026-09-29

**Authors:** Natasha Mootoo, Anya Doorpat, Fariah Karim, Karishma Maharajh, Oluwatomi Oredein, Kegan Romelle Jones

**Affiliations:** 1 Department of Clinical Veterinary Sciences (DCVS), School of Veterinary Medicine (SVM), Faculty of Medical Sciences (FMS), The University of the West Indies (UWI), St. Augustine Campus, St. Augustine, Trinidad and Tobago; 2 Department of Basic Veterinary Sciences (DBVS), School of Veterinary Medicine (SVM), Faculty of Medical Sciences (FMS), The University of the West Indies (UWI), St. Augustine Campus, St. Augustine, Trinidad and Tobago

**Keywords:** antibiotics, fluid therapy, gastroenteritis, hospitalization, multimodal, parvovirosis

## Abstract

This research explored the approaches of veterinarians to diagnosis and treatment of gastroenteritis in puppies up to 1 year of age in Trinidad and Tobago and Barbados. The study assessed clinical decision-making, therapeutic interventions, and utilisation of evidence-based practices. Data were gathered from veterinarians through online surveys and semi-structured interviews. The study revealed a significant association between the diagnosis of parvovirus and hospitalisation (*p* < 0.001), as well as between the diagnosis of parvovirus and intravenous (IV) fluid administration (*p* = 0.002). All veterinarians used a multi-drug regimen in treatment. Similarities in treatment protocols among veterinarians with different years of activity were observed, with the most used therapeutic agents being lactated ringer’s solution (fluid therapy), metronidazole (antibiotic), vitamins (nutraceutical), metoclopramide (antiemetic), and Fortiflora® (probiotic). Based on the data collected, symptomatic treatment modalities consistent with current international literature are widely utilized by the veterinarians in Barbados. However, due to a low response rate, the same cannot be concluded for Trinidad and Tobago.

## 1 Introduction

Gastroenteritis refers to inflammation of the stomach and intestinal tract (1). The term denotes a syndrome with clinical signs of vomiting, anorexia and/or diarrhoea due to gastrointestinal inflammation. Gastroenteritis often causes a sudden onset of vomiting or diarrhoea, and this is termed ‘acute gastroenteritis’ (2). There are numerous causes of gastroenteritis, and the condition should also be differentiated from other diseases with similar signs such as acute liver or renal injury and acute pancreatitis (1). Gastroenteritis is a common yet potentially fatal condition in puppies depending on its severity. One cause of severe gastroenteritis is canine parvovirosis, a condition to which young puppies are more susceptible (3). Timely, appropriate treatment of gastroenteritis is important, especially in severe cases. Intensive treatment, for the purpose of this study, encompasses the hospitalisation of the puppy with the introduction of intravenous (IV) fluids, medical management of other clinical signs presented using a multi-drug regimen, as well as the use of confirmatory diagnostic tests to guide further treatment.

To date, there is no published data on the approaches of veterinarians in the Caribbean region to the management of gastroenteritis in puppies. This study explored how veterinarians in Trinidad and Tobago and Barbados treat gastroenteritis in puppies up to 1 year of age. Furthermore, an algorithm representative of the current approach to treatment in Barbados was created. The aim of the study was to assess veterinarians’ approach to the treatment of gastroenteritis in puppies in Trinidad and Tobago and Barbados.

## 2 Materials and methods

### 2.1 Data collection

This study was observational and retrospective, with a participatory epidemiological approach being employed. The study was therefore based on a survey inquiring into the diagnostic and therapeutic methods used by small animal veterinarians in treating canine gastroenteritis in puppies. Therefore, inclusion criteria for participants were current veterinary practice of small animal medicine in either Trinidad and Tobago or Barbados, and previous treatment of a gastroenteritis case in a puppy up to 1 year of age.

An online survey via Google forms was the chosen method of data collection due to convenience, time constraints, cost effectiveness and ease of dissemination. The survey (see Supplementary data sheet) collected information on the clinical management of a recent case of gastroenteritis in a puppy ≤1 year of age by each responding veterinarian. There were 44 questions in total, distributed under the following major sections: client consent, background [information], signalment, history, deworming, vaccination status and vaccines, diagnosis, treatment and patient outcome. Under these sections, qualitative and quantitative data collected included the responding veterinarian’s practice type (clinic-based or mobile), the patient’s presenting clinical signs, vaccination and deworming status of the patient, clinical signs and diagnostic methods used to assist in arrival at the diagnosis (inclusive of Parvo ELISA testing, faecal examination, complete blood count (CBC), radiography, ultrasonography, palpation, and other diagnostic procedures), the cause of gastroenteritis as identified by the veterinarian, treatment practices (including hospitalization, fluid therapy protocols, dietary management, administration of antibiotics, analgesics, antiemetics, appetite stimulants, nutraceuticals (including probiotics and vitamins), patient outcome (survival), and the number of years of activity of the veterinarian.

The target population for the survey was small animal veterinarians across Trinidad and Tobago and Barbados. For Barbados, a list of registered veterinarians was obtained from the Barbados Government Veterinary Services. This list included 70 veterinarians. Veterinarians that had relocated to another country, as well as those who indicated to the authors that they would be unable to complete the survey (for reasons including being retired, not practising small animal medicine, not having gastroenteritis cases) were ineligible for the study and hence removed from the list. Veterinarians that were deceased were also removed from the list. Veterinarians whom the authors were unable to contact remained on the list. This left 43 veterinarians, which were included in the study population. Veterinarians whom the registered list had not yet been updated to include were also included in the study population. Hence, the total study population in Barbados was 47 veterinarians. According to the Trinidad and Tobago Veterinary Association (TTVA) there were a total of 479 registered veterinarians in Trinidad and Tobago as of 2023 (4). However, according to the UWI SVM Exit Survey (5) conducted in 2022, only 65% of the veterinarians practice small animal medicine. The assumption was made that all practising small animal veterinarians would have treated a case of gastroenteritis in a puppy. Hence, in Trinidad, the study population consisted of approximately 311 veterinarians. The total study population for the project was therefore determined to be 358 (47 + 311) veterinarians.

A sample size calculator (6), utilising the equation, sample size (*n*) = [(*z*^2^ × *p*(1 − *p*)) ÷ *e*^2^] ÷ [1 + ((*z*^2^ × *p*(1 − *p*)) ÷ *e*^2^*N*)], where *z* = *z*-score = 1.96 (based on the 95% confidence level) (7), *p* = standard deviation (assuming to be 0.5), *e* = margin of error and *N* = population size, was used to compute the sample size of veterinarians for this study. The confidence level was set at 95% thus making the error rate 5%. Using this equation, for Barbados (*N* = 47), a sample size of 42 veterinarians was calculated. For Trinidad (*N* = 311), a sample size of 173 veterinarians was calculated. Hence, the total sample size was 215 (173 + 42) veterinarians.

*For Barbados, inability to contact some veterinarians on the list precluded confirmation of their status (i.e., whether they met the inclusion criteria). Including them in the study population may therefore have resulted in a larger (than the actual) study population. Similarly, for Trinidad, demarcating all small animal practitioners as the study population may have resulted in veterinarians that did not meet the inclusion criterion (of having treated a gastroenteritis case) being included in the study population. For both countries, this could have led to calculation of a larger (than true) sample size. Despite this, concern that ineligible persons (those not meeting the inclusion criterion) may have entered the study was virtually eliminated, as the survey was based on the last case of puppy gastroenteritis that a veterinarian had treated.

Non-probability sampling, specifically convenience sampling, was conducted to attain proper case records. A link to the survey was shared to veterinarians via email, social media and professional networks (the Trinidad and Tobago Veterinary Association and Barbados Veterinary Association). The survey was opened in June 2024 and closed in December 2024, with reminders sent to veterinarians during the period to maximize participation. Veterinarians completed the survey online at their convenience. The survey was also conducted in-person via semi-structured interviews at various clinics in the counties and parishes of Trinidad and Tobago and Barbados, respectively. The interviews followed the same structured questionnaire as the online survey to ensure consistency and comparability of responses, with minor probing questions used where necessary for clarification. Interviewee responses were entered into the Google Forms survey by the interviewer in real time.

Ethical approval for this project was granted by the Campus Research Ethics Committee of the University of the West Indies, St. Augustine campus.

### 2.2 Statistical analysis

The qualitative and quantitative data collected under the above-mentioned categories (see section 2.1) were automatically entered into an Excel spreadsheet linked to the Google forms survey. The data was then formatted in Excel for export into the SPSS program where inferential and descriptive statistical analyses were performed on selected variables. Inferential statistics consisted of Pearson Chi- Square tests to establish associations among our variables, while the descriptive statistics of mode, frequencies and cross-tabulations were used to summarize data.

Chi-square tests were performed on the following variables:

- Diagnosis of parvovirus (derived from survey q. 19) and Hospitalization (q. 22)
- Diagnosis of parvovirus and Use of IV fluids (q. derived from q. 24)
- Use of intensive treatment* and Less than 10 years of activity (derived from q. 5)

Intensive treatment is defined in Section 1 (introduction). This compound, dichotomous variable was derived as follows; subjects with a ‘yes’ value for the derived, dichotomous variables of IV fluid use [derived from q. 24], use of more than one drug, and use of confirmatory diagnostic tests [derived from q. 21] were assigned a ‘yes’ value for intensive treatment, i.e., were considered to have utilized intensive treatment as defined in this study. Subjects with a ‘no’ value for any of those three variables were assigned a ‘no’ value for intensive treatment).

Some veterinarians indicated the use of a particular diet in response to the ‘which nutraceuticals’ (q. 41) and ‘other treatment(s)’ (q. 43) questions. These responses were used to derive a dichotomous ‘dietary modification’ variable, with those reporting a diet (e. Purina Proplan EN) assigned a ‘yes’ value, i.e., used dietary modification, and all other subjects assigned a ‘no’ value. i.e., did not use dietary modification.

Frequencies and mode were obtained for the following variables: [which] fluids (q. 24), antibiotics (q. 29), analgesics (q. 27), antiemetics (q. 31), nutraceuticals (derived variable; responses consisted of probiotics, vitamins, iron supplements and other nutraceuticals, i.e., q. 32, 34, 38, 40 combined), probiotics (q. 33), vitamins (q. 35) and appetite stimulants (q. 37) were administered. The frequencies for type of dietary modification used were obtained via manual count of responses in the excel spreadsheet.

Crosstabulations were done between the types of a particular treatment used (e.g., q. 24: which fluids administered) and the number of years of activity of the veterinarian (q. 5), for the following variables: [which] fluids, antibiotics, analgesics, antiemetics, nutraceuticals (consisting of probiotics, vitamins, iron supplements and other nutraceuticals) probiotics, vitamins and appetite stimulants were administered.

The statistical findings were then displayed [see Section 3. Results] using charts and graphs to highlight patterns and anomalies. An algorithm was also created to represent data from Barbados based on the analyses.

Note: “q. x” = Question no. x in the survey (in Supplementary data sheet).

## 3 Results

A total of 88 veterinarians (58 from Trinidad and Tobago, and 30 from Barbados) participated in the online survey on the last gastroenteritis case they treated in a puppy 1 year old or younger. The majority of veterinarians were clinic-based, while a smaller percentage (12.5% i.e., 11/88) were mobile veterinarians (Supplementary data sheet q. 3–4).

Clinical signs and findings reported (q. 10) included vomiting, non-haemorrhagic diarrhoea, haemorrhagic diarrhoea, foul odour with diarrhoea, fever, appetite loss, lethargy and/or depression, with numerous different combinations of these reported. The reported historical duration of vomiting, diarrhoea or loss of appetite (q. 12) did not exceed 6 days in any case. The dehydration status of patients was also obtained (q. 11). 60.2% (53/88) were ≥5% dehydrated. Other responses included values between 2 and 4% (5/88), “<5%” (5/88), adequately hydrated/not dehydrated/0% (7/88), and unknown (18/88).

Methods aiding in diagnosis of the cause of gastroenteritis included the use of history (inclusive of clinical signs), and various diagnostic tests (Supplementary data sheet; q. 13, 20, 21). Statement of other historical findings aiding in diagnosis was not required by the survey. A diagnostic test for parvovirus was performed by 53.4% (47/88) of veterinarians, and 12.5% (11/88) of veterinarians conducted a faecal examination (faecal smear and/or faecal flotation). Furthermore, approximately 8% (7/88) conducted both a parvovirus test and faecal examination (Figure 1). The majority of respondents (41/47) utilizing a parvovirus test selected the “Parvo ELISA Test” option provided by the survey (q. 21). One respondent specifically reported use of the IDEXX Parvo snap test (an ELISA assay). The remaining tests (5/47) used in diagnosing/ruling out parvovirus were a “CPV Antigen Test” (1/5), a Canine Parvovirus + Canine Coronavirus + Giardia lateral flow immunochromatographic assay (CPV + CCV + Giardia LFIA) (2/5), “Abaxis parvo rapid test” (1/5) and a “parvo rapid test” (1/5). Note that the results of the diagnostic tests for parvovirus were not obtained by the survey. The information obtained was whether a diagnostic test aided in arrival at the definitive diagnosis (q. 21). For those reporting CPV as the cause of enteritis (q. 19), along with use of a diagnostic test for CPV as aiding their definitive diagnosis (q. 21), the assumption was made that the test for parvovirus was positive. Other diagnostic tests reported included SNAP Giardia test, 4dx PLUS SNAP Test, complete blood count (CBC), chemistry 18 panel, (SDMA) radiography, ultrasonography and palpation.

**Figure 1:**
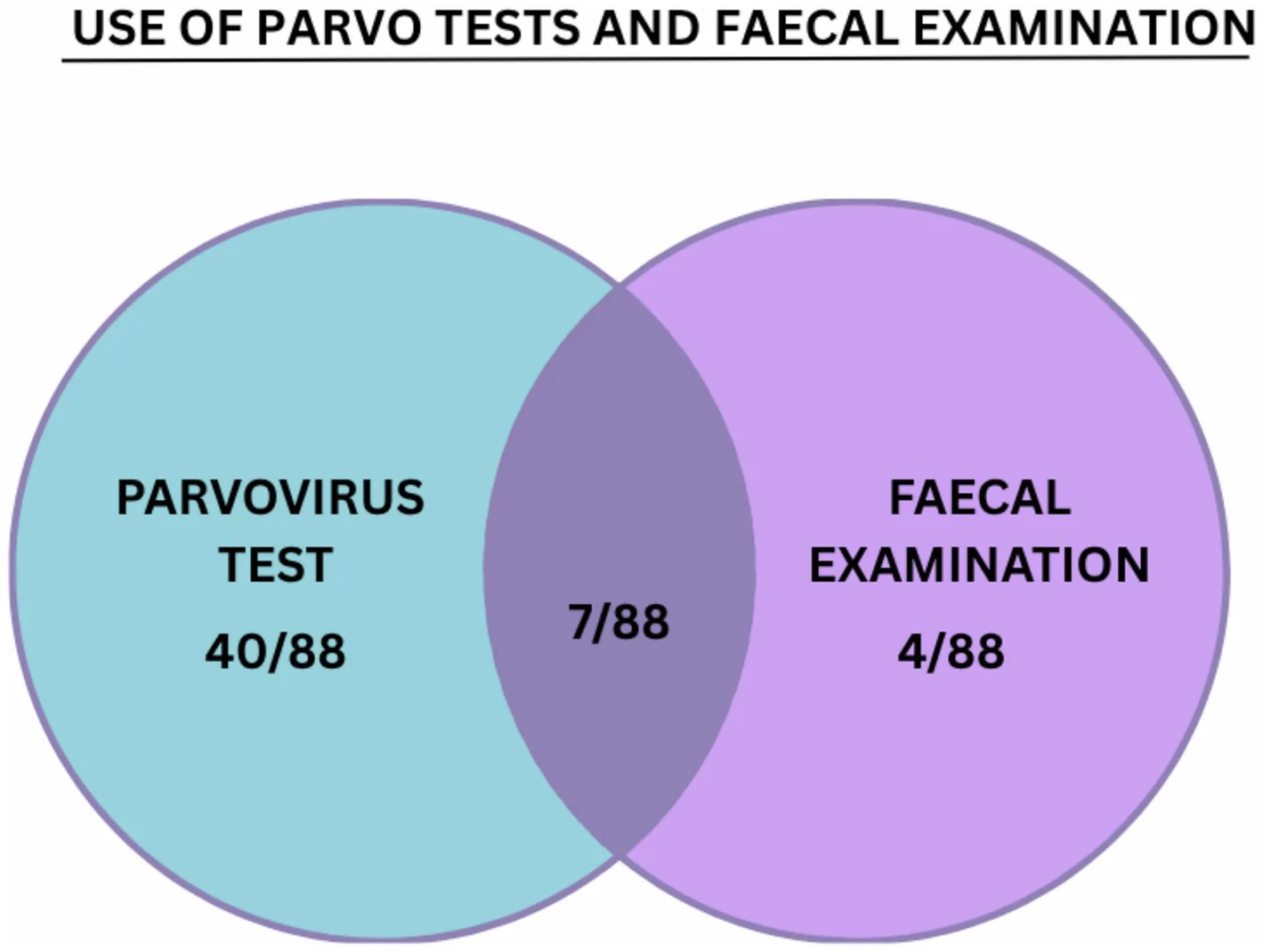
Use of diagnostic tests for parvovirus and faecal examination.

The most commonly diagnosed cause of gastroenteritis (q. 19) was parvoviral enteritis, accounting for 72.7% (64/88) of cases. The second most common cause was a bacterial infection 17% (15/88) and the third, endoparasitism 10.2% (9/88). Unknown causes accounted for 6.8% (6/88) of the cases, while a polyp and a foreign body each made up 1.1% (1/88) of the cases. For some cases, more than one cause of gastroenteritis was diagnosed.

The Chi-Square Test of Independence was utilized to assess whether an association existed between the diagnosis of parvoviral enteritis and hospitalization (q. 22), and between the diagnosis of parvoviral enteritis and IV fluid administration (q. 24). There was a significant association between a parvoviral enteritis diagnosis and hospitalization (*p <* 0.001), as well as between diagnosis of parvoviral enteritis and administration of IV fluids (*p* = 0.002) (Table 1).

**Table 1:** Parvo diagnosis vs. hospitalization and IV fluids.

	*N*	Pearson chi-square value	*p*-value^*^
Hospitalization	88	11.853	<0.001
IV fluids	88	9.734	0.002

^*^The chosen level of significance (*α*) was 0.05.

The majority of veterinarians, i.e., 81.8% (72/88) of veterinarians administered fluids to their patient, with 73.9% (65/88) of veterinarians utilizing the intravenous route of administration (Supplementary Figure 1).

Lactated Ringer’s solution (LRS) was the most frequently used fluid with 73.6% (53/72) of veterinarians using it in the treatment of gastroenteritis in puppies up to 1 year of age. Subsequently preferred by veterinarians was 0.9% saline with 34.7% (25/72) followed by 5% dextrose in 0.9% saline with 22.2% (16/72) of veterinarians using it (Figure 2). In some cases, multiple fluids were administered.

**Figure 2:**
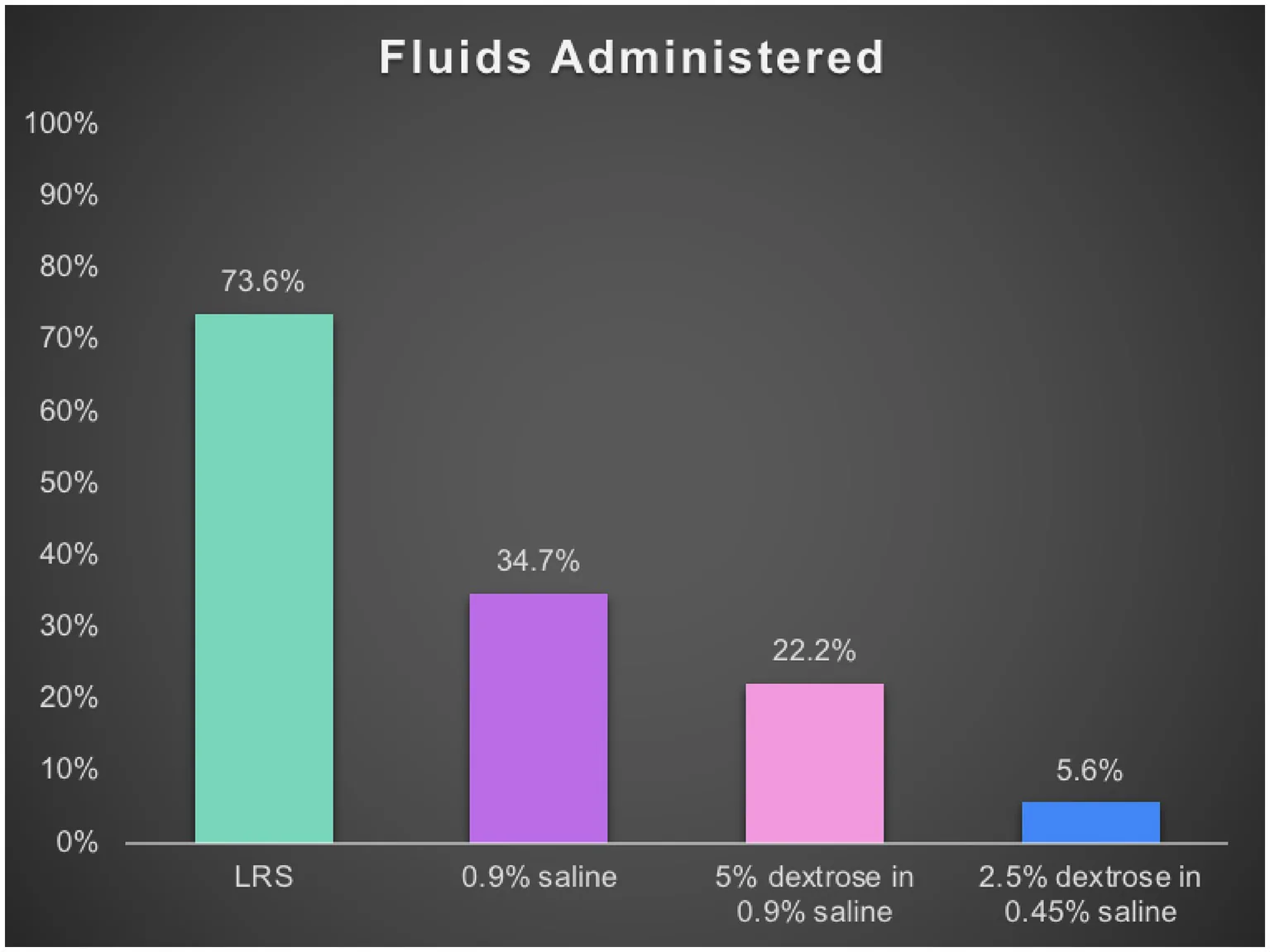
Fluids used by veterinarians in T&T and Barbados.

Eighty-eight percent of veterinarians (77/88) did not report dietary modification as part of treatment of their gastroenteritis case. Seven percent (6/88) used PurinaⓇ Pro PlanⓇ EN (Gastroenteric Nutrition) and 4/88 PurinaⓇ Pro PlanⓇ CN (Critical Nutrition). Lastly, 1% of veterinarians utilized a Bland Diet (Figure 3).

**Figure 3:**
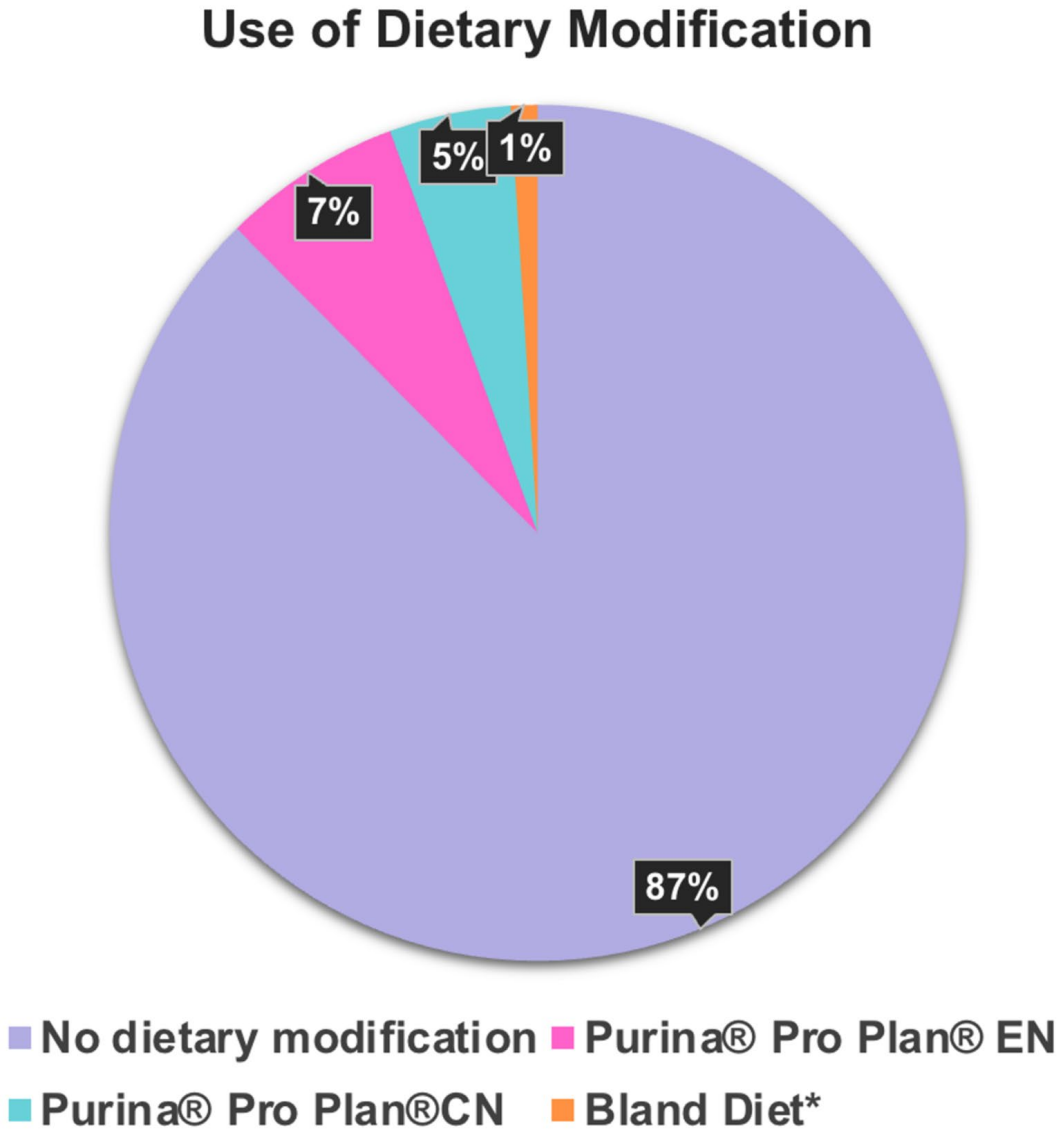
Use of dietary modification by veterinarians *bland diet, i.e., “rice and chicken/eggs without salt or seasoning”.

Approximately 93% (82/88) of veterinarians used broad spectrum antibiotics while 7% of veterinarians (6/88) did not use any antibiotic therapy (Figure 4).

**Figure 4:**
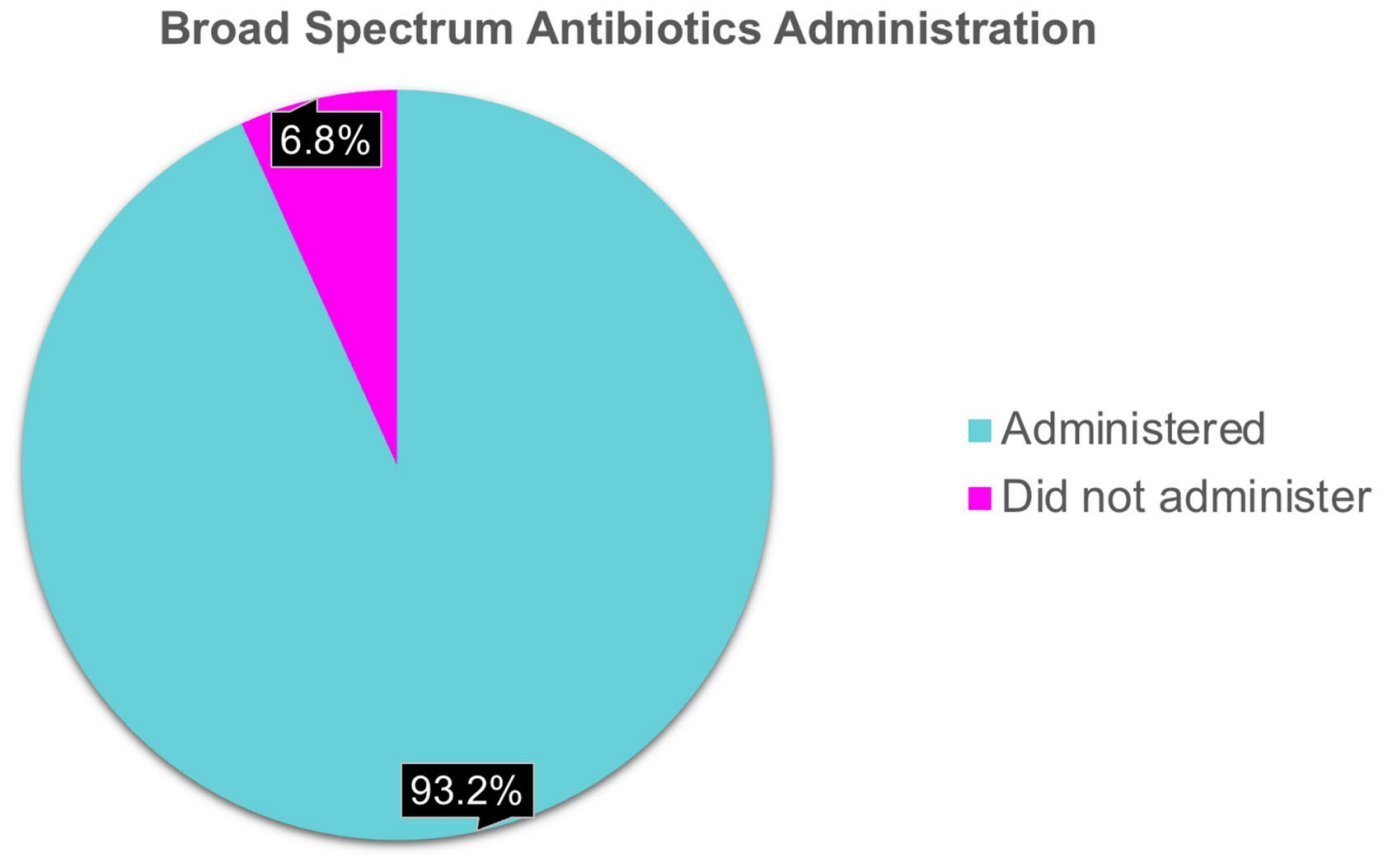
Broad spectrum antibiotics used by veterinarians in T&T and Barbados.

Metronidazole was the most frequently used antibiotic with 51.2% (42/82) veterinarians using it in the treatment of gastroenteritis in puppies up to 1 year of age. Subsequently preferred by veterinarians was amoxicillin (35.4% (29/82) followed by trimethoprim/ sulfamethoxazole (24.4% i.e., 20/82 veterinarians). In some cases, multiple antibiotics were used (Figure 5).

**Figure 5:**
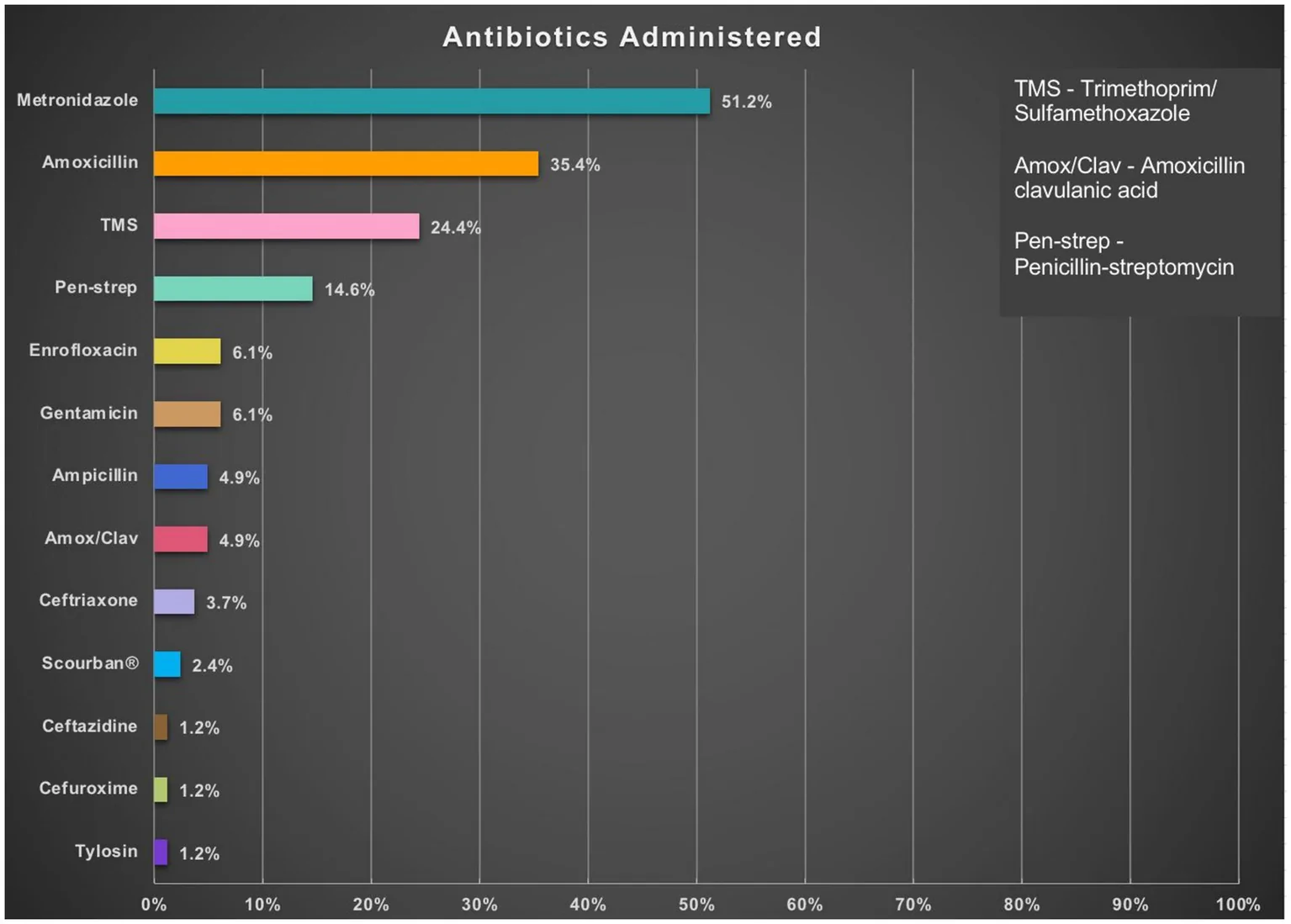
Antibiotics used by veterinarians in T&T and Barbados. Scourban® contains sulfadimidine, sulfadiazine, streptomycin and neomycin.

Meloxicam was the most frequently used analgesic with 66.7% (18/27) of veterinarians including it in their treatment. Subsequently preferred by veterinarians was tramadol used by18.5% (5/27), followed by flunixin (14.8% (4/27) In one case, both meloxicam and tramadol were used (Figure 6).

**Figure 6:**
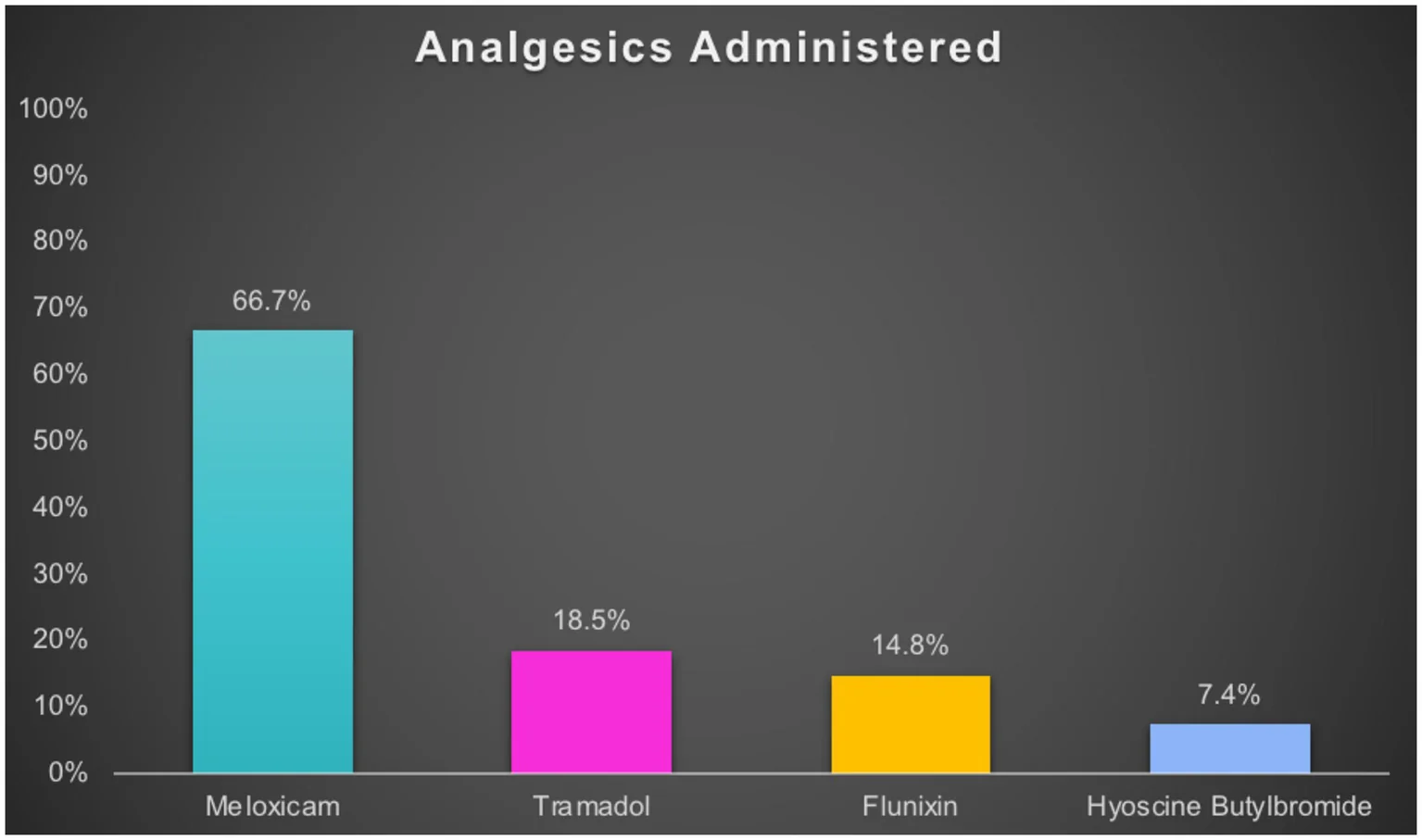
Analgesics used by veterinarians in T&T and Barbados.

Metoclopramide was the most frequently used antiemetic, with 55.6% (40/72) of veterinarians including it in treatment of their gastroenteritis case. Subsequently preferred by veterinarians was maropitant (41.7% (30/72), followed by dimenhydrinate (11.1% (8/72) of veterinarians) (Figure 7). In some cases, more than one antiemetic was administered.

**Figure 7:**
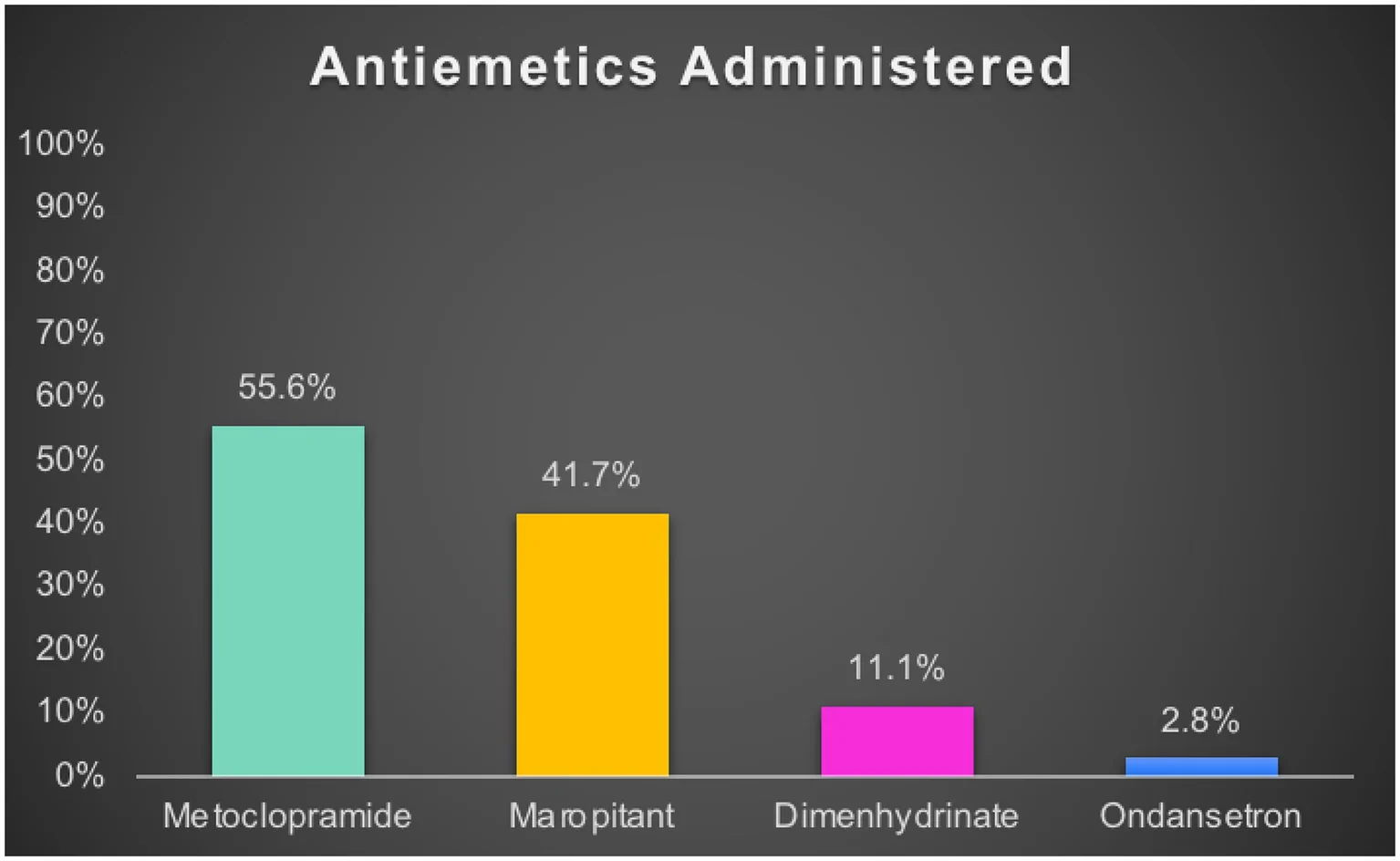
Antiemetics used by veterinarians in T&T and Barbados.

A total of 76% (19/25) of veterinarians administered cyproheptadine making it the most commonly used appetite stimulant. Mirtazapine was the second most used (12% i.e., 3/25) followed by B-complex (4% i.e., 1/25) In some cases, more than one appetite stimulant was used (Figure 8).

**Figure 8:**
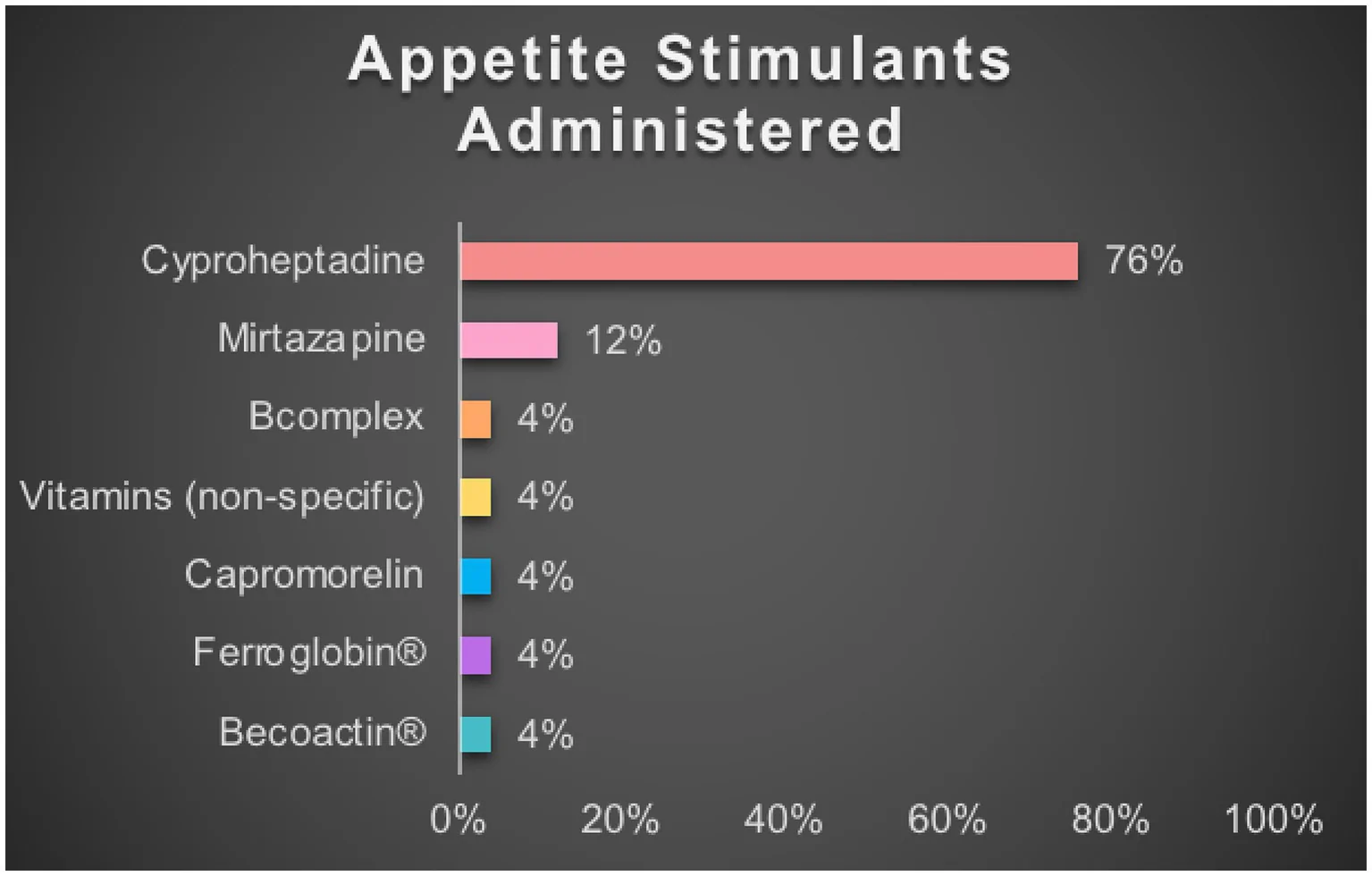
Appetite Stimulants used by veterinarians in T&T and Barbados.

Sixty-eight (i.e., 77%) of the veterinarians administered nutraceuticals as part of their treatment. Of these, 58/ i.e., 85.3% veterinarians utilized vitamins, making it the most used nutraceutical. The second most used was probiotics 52.9% (35/68), followed by various “other nutraceuticals” 26.5% (18/68) (Figure 9).

**Figure 9:**
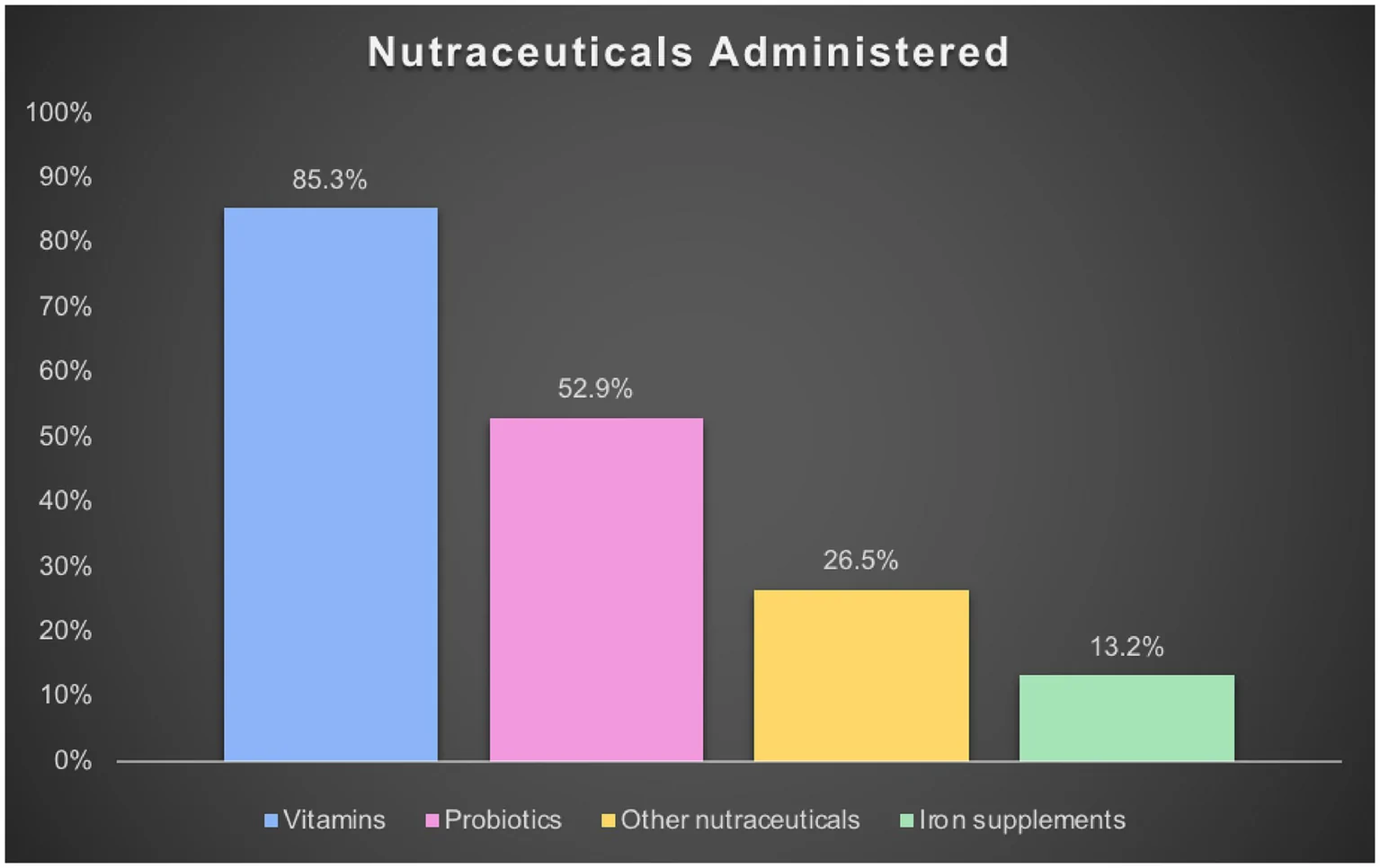
Nutraceuticals used by veterinarians in T&T and Barbados. _Other nutraceuticals included theanine adjuvants, Purina® Pro Plan® Critical Nutrition, Purina® Pro Plan® Gastroenteric Formula, cat’s claw, coconut water, aminolean, rehydration salts and Ensure®_.

“Other nutraceuticals” included theanine adjuvants, Purina® Pro Plan Critical Nutrition®, Purina® Pro Plan Gastroenteric Formula, Cat’s claw, coconut water, aminolean, rehydration salts and Ensure®.

The majority, i.e., 41.4% (24/58) of veterinarians which gave vitamins administered multivitamins, (Supplementary Figure 2). Vitamin B12 was the second most used 32.8% (19/58) followed by B complex 25.9% (15/58).

Of the veterinarians administering probiotics, 63.9% (23/36) gave Fortiflora®, making it the most commonly used probiotic for treating gastroenteritis in puppies. The second most used was yogurt 19.4% (6/36) and the third most used was Enterogermina® (11.1% i.e., 3/36) (Supplementary Figure 3).

The number of years of activity among the veterinarians participating in this study ranged from 0 to 45. Sixty percent (60%) of veterinarians had 0–10 years of activity in the field, 35% had 11–25 years of activity and 5% had 26–45 years of activity (Supplementary Figure 4).

Note that these years of activity ranges were further divided into 5-year categories for cross-tabulation analyses (see Supplementary Figures 5a–10b).

LRS was the most commonly used fluid among veterinarians across all the years of activity categories (Supplementary Figures 5a,b).

Metronidazole was the most used antibiotic among veterinarians with 0–5, 6–10, 11–15 and 16–20 years of activity (Supplementary Figures 6a,b). Amoxicillin was the mode for veterinarians with 21–25 years of activity (Supplementary Figure 6c). There was no mode for antibiotic use by veterinarians with 26–30, 36–40 and 41–45 years of activity (Supplementary Figures 6c,d).

Meloxicam was the most frequently used analgesic among veterinarians with 0–10 years (x/x, x%) and 16–20 years of activity, while flunixin was the most frequently used analgesic among veterinarians with 11–15 years of activity (Supplementary Figure 7). It should also be noted that analgesics were not used by veterinarians with 21–45 years of activity.

Metoclopramide was the most commonly used antiemetic among veterinarians with 0–5, 6–10, 21–25 and 36–40 years of activity, while maropitant was the most commonly administered among veterinarians with 11–15 and 16–20 years of activity. Veterinarians with 41–45 years of activity did not use antiemetics (Supplementary Figures 8a,b).

Fortiflora® was the most frequently administered probiotic among veterinarians with 0–5, 11–15, 16–20 and 21–25 years of activity. Additionally, it was observed that yogurt was the most commonly used probiotic among veterinarians with 6–10 years of activity. Notably, no probiotics were used among veterinarians with 36–45 years of activity (Supplementary Figures 9a,b).

Vitamins were the most frequently used nutraceutical among veterinarians with 0–5, 6–10 and 16–20 years of activity. Both vitamins and probiotics were the most used among veterinarians with 11–15 and 21–25 years of activity. Probiotics were the most frequently used nutraceutical for veterinarians with 21–25 years of activity. (Supplementary Figures 10a,b) Nutraceuticals were not used among veterinarians with 26–45 years of activity.

The Chi-Square Test of Independence was done to determine if an association existed between the use of intensive treatment (defined in Introduction) of gastroenteritis and a veterinarian having less than 10 years of activity in the field. A *p*-value of 0.787 was obtained, indicating that there was not a significant association between the use of an intensive approach and a veterinarian having less than 10 years of activity (Table 2).

**Table 2:** Use of intensive treatment vs. less than 10 years of activity.

	*N*	Pearson chi-square value	*p*-value^*^
Intensive treatment	87	0.073	0.787

^*^The chosen level of significance (*α*) was 0.05.

An algorithm was created to demonstrate the treatment of gastroenteritis in Barbados (Figure 10). Although Trinidad and Tobago had more responses in absolute terms (58/88), the lower response rate for its calculated sample size (58/173, i.e., 33.5%) was insufficient to be representative and therefore did not support development treatment algorithm for that country. Barbados, despite fewer responses of the total obtained (30/88), had a higher response rate (30/42, i.e., 71.4%), making the findings more representative of clinical practice in the country. To generate this algorithm, only the responses from Barbados were used. This is in contrast to all other findings and analyses reported, for which all 88 responses were used. Under the “Gastroenteritis” box are the three causes of gastroenteritis (both definitively and presumptively diagnosed) reported by veterinarians in Barbados. The arrow from each cause goes to any treatment method that was utilised by more than 50% of veterinarians choosing that cause. Then the following arrow goes to the most common drug used for each treatment method. The chart also highlights the usage of additional supportive therapies in “Other Treatments,” particularly for parvoviral enteritis; in parentheses next to each therapy is the percentage of veterinarians (diagnosing parvoviral enteritis) that used this therapy. It is important to clarify that this algorithm is purely a display of current practices in the country, and not a prescriptive algorithm recommending what should be done.

**Figure 10:**
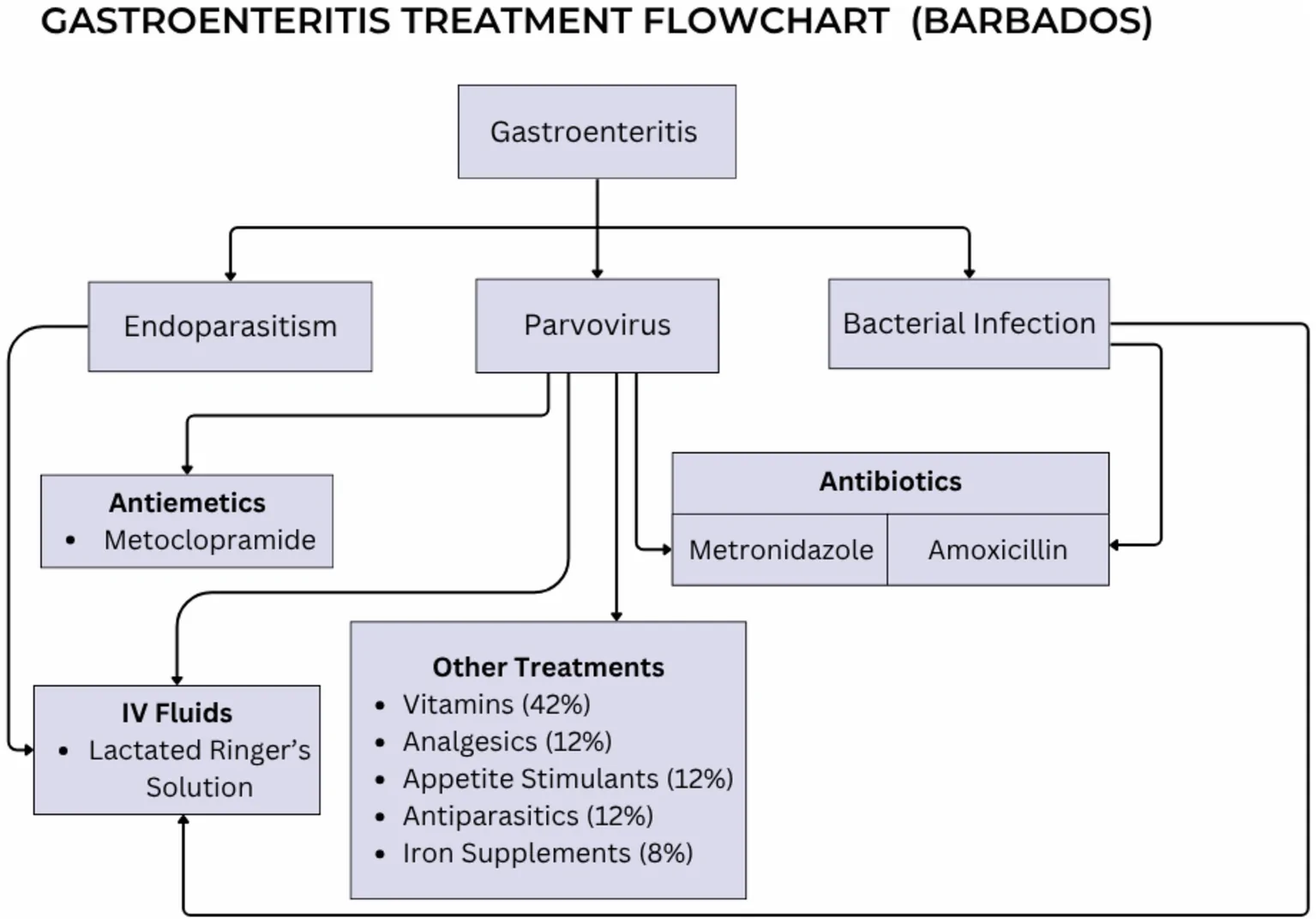
Treatment of gastroenteritis in Barbados.

## 4 Discussion

This study explored the approach of veterinarians in Trinidad, Tobago, and Barbados to the treatment of gastroenteritis in puppies. It revealed an association between the diagnosis of parvoviral gastroenteritis in puppies and two main treatment strategies, i.e., IV fluid administration and hospitalization. Treatment options that are typically used in the regimen for gastroenteritis in puppies were also investigated and will be discussed. The clinical signs of gastroenteritis vary little despite the underlying cause, and include vomiting, diarrhoea and anorexia. Addressing these clinical signs and their implications through symptomatic therapy is usually sufficient for resolution of the condition, and in fact, the specific cause of gastroenteritis is not often identified (1). The treatment options explored in this retrospective study are mainly those which address the clinical signs of gastroenteritis. While many experimental studies have explored the efficacy of various treatments in managing canine gastroenteritis (7–9), fewer studies have observationally explored the approach of veterinarians to managing this condition or related entities. One study in the UK explored the clinical management of dogs with uncomplicated acute diarrhoea; cases of diarrhoea within a broader diagnosis such as confirmed infection were not included (10). Another study in Denmark explored the management and outcome of suspected Acute Haemorrhagic Diarrhoea Syndrome (AHDS) in dogs (11). This current study explored clinical management of the broader condition of gastroenteritis, in puppies, including cases with diagnosed aetiologies.

Diagnoses of the cause of gastroenteritis were mainly made using clinical signs, history and various diagnostic tests. The authors did not inquire which specific historical findings informed the veterinarian’s diagnosis The most commonly diagnosed cause of gastroenteritis was Parvoviral enteritis (64/88, i.e., 72.7%) which was diagnosed by the majority of veterinarians (40/64, i.e., 62.5%), using a diagnostictest for Parvovirus (i.e., ELISA Test; 36/40 vets, CPV + CCV + Giardia LFIA; 2/40, Abaxis parvo rapid test; 1/40 or “Parvo rapid test”; 1/40). The remaining 25 veterinarians presumptively diagnosed parvoviral enteritis using clinical signs /clinical signs and history. Haemorrhagic diarrhoea and a foul odour with diarrhoea, common signs of parvoviral enteritis (12), were noted in 22/25 (88%) and 15/25 (60%) of these cases, respectively. Bacterial infection was the second most common diagnosis (15/88) made by veterinarians. Thirty-three percent (5/15) stated a complete blood count (CBC) as one of the tests aiding their arrival at this diagnosis. Bacterial enteritis in canines can be diagnosed using fecal culture, PCR testing, and toxin assays alongside clinical signs like diarrhoea, vomiting, fever, and abdominal pain. However, these clinical signs are not specific to a bacterial enteritis (13). Fecal culture can identify organisms such as *Salmonella* spp. and *Campylobacter* spp., while PCR is sensitive for detecting bacterial DNA and the presence of toxins in feces. ELISA or toxin assays may also be used to detect *Clostridium difficile* or *Clostridium perfringens* toxins (13, 14).

While a complete blood count (CBC) cannot definitively diagnose bacterial enteritis in dogs or puppies, it can support the diagnosis when interpreted with clinical signs and fecal testing (13). Common CBC findings include leukocytosis with neutrophilia, a left shift (increased immature neutrophils), and toxic neutrophil changes such as Döhle bodies or cytoplasmic basophilia, which indicate acute bacterial inflammation. Severe infections may also cause neutropenia with a degenerative left shift (more immature neutrophils than mature neutrophils) if neutrophil demand exceeds bone marrow supply (15). The specific CBC parameters assessed by those diagnosing bacterial infection were not captured in this study. Endoparasitism was the cause of gastroenteritis in 9/88 cases. 2/9 cases were confirmed using faecal flotation and 1/9 cases with a SNAP Giardia Test. It should be noted that sometimes concurrent causes of gastroenteritis were diagnosed such as Parvovirus and bacterial infection. In one case, a polyp was the cause of gastroenteritis and this diagnosis was confirmed using ultrasonography. Another case was diagnosed as a foreign body and x-ray was used to confirm this diagnosis. The remaining cases of gastroenteritis (6/88) were reported as having an unknown cause.

The predominance of parvoviral enteritis in the present study is consistent with previous studies identifying canine parvovirus as one of the leading causes of acute gastroenteritis in puppies. A molecular survey reported Parvoviral enteritis in 64.8% of canine gastroenteritis cases, similarly demonstrating the predominance of parvoviral infection in young dogs (16). The frequent use of Parvo ELISA tests by veterinarians in the present study also agrees with previous literature reporting ELISA as one of the most commonly utilized diagnostic methods due to its high specificity and practicality (17). Concurrent diagnoses of parvoviral and bacterial enteritis observed in this study are also supported by previous reports documenting co-infections involving viral, bacterial and parasitic enteropathogens in dogs with diarrhoea (18) Figure 1 illustrates that 53.4% (47/88) of veterinarians performed a diagnostic test for parvovirus while 12.5% (11/88) performed faecal examination (faecal smear and/or faecal flotation), whereas 8% (7/88) of veterinarians performed both tests. The predominance of using Parvo ELISA tests (41/47 veterinarians) can be attributed to their high specificity ≥95% in some test kits (19, 20), convenience and ability to provide results within minutes (21), enabling veterinarians to make timely decisions regarding treatment and patient care. Faecal examination may have been utilized less as many veterinarians may not have suspected endoparasitism to be the primary cause of gastroenteritis. Veterinarians routinely deworm puppies prior to and as part of their protocol, it may be assumed that if the puppy was seen before or was recently dewormed, a high endoparasite burden is less likely to be the primary cause of diarrhoea. Eight percent (8%) of veterinarians employed both diagnostic methods, perhapsto allow for detection of both endoparasitism and parvoviral infection as possible causes of gastroenteritis for suspected cases of high gastrointestinal worm burdens or persistent endoparasitism due to possible drug resistance.

Table 1 showed that there was a significant association (*p* = 0.002) between the diagnosis of parvoviral enteritis and the inclusion of intravenous (IV) fluids as part of their treatment regimen. Although this chi-square test result only proved an association between the two variables, it can be postulated that it was the diagnosis of parvoviral enteritis that influenced the decision to use IV fluids. This may suggest that IV fluid therapy is recognized as a critical component of supportive care for parvoviral enteritis, likely due to the severe dehydration, electrolyte imbalances and in some cases hypovolemic or septic shock associated with the disease. This emphasizes the importance of fluid resuscitation in clinical management of parvoviral enteritis (22). A significant association was also observed between the diagnosis of parvoviral enteritis and hospitalization (*p* < 0.001) as shown in Table 1. It can again be surmised that the diagnosis of parvoviral enteritis influenced the decision to hospitalize the patient. This may suggest that veterinarians in the islands studied prioritize inpatient care for severely affected puppies, and aligns with the understanding that parvoviral enteritis requires intensive supportive care which is more easily provided in an inpatient rather than an outpatient setting. Management of parvoviral enteritis includes continuous IV fluids, antiemetics, broad-spectrum antibiotics, nutritional support (22), close monitoring, and isolation to prevent disease spread. In essence, veterinarians must employ an intensive, multimodal approach to the management of this condition. The result of the chi-square test reinforces the notion that hospitalization is considered essential for improving survival rates in parvovirus cases. However, one study (23) showed that there was not a statistically significant difference between survival rates for parvovirus patients receiving standard in-hospital treatment versus an outpatient protocol In another study, the survival rate for patients (with a known outcome) on an outpatient protocol was 74% (24), similar to the 80% in the previously mentioned study (23).

For the Chi-square tests assessing associations between the diagnosis of parvovirus and hospitalization or IV fluids, all parvoviral enteritis diagnoses were included, whether definitive or presumptive. The diagnosis of parvovirus via a test (see results section; paragraph describing Figure 1) assay was considered definitive, while presumptive diagnosis was based on clinical signs, history, and other supportive laboratory tests. While the occurrence of both definitive and presumptive diagnosis is reflective of clinical practice, this distinction is important when interpreting the observed associations. Presumptive diagnoses may be subject to error, particularly where other causes of gastroenteritis present similarly. As such, their inclusion may have influenced the observed associations with interventions such as intravenous fluid therapy and hospitalization, which may partly reflect clinician response to disease severity rather than confirmed infection. Future studies with greater use of confirmatory diagnostics may allow for more precise evaluation of these relationships.

Gastroenteritis patients have been observed to respond well to food being withheld for 24–48 h in order to rest the GIT. Food is then slowly reintroduced beginning with wet, easily digestible food (1). However, early enteral nutrition prevents intestinal atrophy, has been shown to maintain intestinal integrity during acute diarrhoea (25), and may reduce the increased intestinal permeability that occurs due to inflammation in an acute gastroenteritis patient (7). Hence, early enteral nutrition can be a suitable alternative to initial withholding of food for one to two days in acute gastroenteritis. Figure 2 illustrates that 88% of veterinarians (77/88) did not implement dietary modification. This may suggest that some veterinarians may not view dietary change as essential for treatment of gastroenteritis, or they may prioritize other treatment modalities such as fluids, antiemetics and may consider dietary changes when the patient stabilizes. In dogs that are vomiting subsequent to gastritis, food should be highly digestible to reduce the amount of hydrochloric acid (HCl) released in the stomach, as higher amounts of HCl may cause further mucosal damage to an already inflamed gastrointestinal tract (26). Twelve percent of veterinarians (11/88) instituted dietary modification. One percent utilized a ‘bland diet’ (which was specified to be rice and chicken/eggs without salt or seasoning). A bland diet refers to a diet that is easily digestible, low in fat, mildly flavored, and non-irritating to the gastrointestinal (GI) tract. Common components include highly digestible carbohydrates such as white rice and boiled potatoes, and lean high biological value protein (HBV) sources such as boiled skinless chicken, turkey or cottage cheese (27, 28). Feeding a puppy food with HBV protein is crucial for recovering from gastroenteritis as high-quality proteins are highly digestible (29). HBV protein refers to protein that contains essential amino acids in proportions efficiently utilized by the body for tissue synthesis and repair (30). Animal-derived proteins such as eggs, chicken, fish, and milk proteins are considered HBV proteins (29). In puppies with gastroenteritis, HBV proteins are beneficial because they support intestinal mucosal repair, improve nitrogen balance, reduce muscle catabolism, and provide essential amino acids necessary for growth and immune function during illness (31). Also, bland diets are commonly recommended in puppies with gastroenteritis because intestinal inflammation impairs digestion and absorption (29). Feeding easily digestible foods helps decrease gastrointestinal workload, maximize nutrient absorption, minimize vomiting and diarrhoea, and support intestinal healing while maintaining caloric intake and hydration status (28).

Bland diets of boiled chicken and rice have traditionally been recommended to provide a simple, easily digestible meal for dogs experiencing gastrointestinal upset. However, research in veterinary nutrition has led to the development of high-quality prescription diets that are more beneficial, as they are formulated to support gastrointestinal health by providing a bland yet nutritionally complete diet as it includes necessary vitamins and minerals (32). In this study, 7 % of veterinarians (6/88) used PurinaⓇ Pro PlanⓇ EN (Gastroenteric Nutrition) and 5 % (4/88), PurinaⓇ Pro PlanⓇ CN (Critical Nutrition). Purina Pro Plan EN is an easily digestible diet and promotes digestive health (33). Prescription gastrointestinal diets such as PurinaⓇ Pro PlanⓇ EN are formulated to support recovery through the use of highly digestible protein sources, moderate fat content, and balanced nutrient profiles that reduce digestive workload while maintaining adequate caloric intake. Highly digestible proteins improve nutrient absorption and reduce the amount of undigested substrate reaching the intestine, thereby helping to minimize osmotic diarrhoea and intestinal fermentation. Appropriate dietary lipid content is also important, as excessive fat may delay gastric emptying and worsen vomiting or malabsorption in some patients, whereas moderate fat levels help meet energy requirements during recovery (34).

Several studies evaluating dietary management in canine gastroenteritis have emphasized the benefits of nutritional intervention during recovery. In a randomized clinical trial involving dogs with severe parvoviral enteritis, early enteral nutrition administered via nasoesophageal tube was associated with improved intestinal permeability, reduced intestinal protein loss, and earlier clinical recovery compared with withholding food until vomiting ceased (7). Similarly, another reported that young dogs with haemorrhagic gastroenteritis receiving combined parenteral and early enteral nutrition demonstrated improved metabolic stability and better maintenance of gastrointestinal function compared with dogs receiving parenteral nutrition alone (28).

More recent nutritional studies have also evaluated the role of specialized highly digestible gastrointestinal diets in dogs with acute diarrhoea. A randomized double-blinded study found that dogs fed a highly digestible prescription diet supplemented with prebiotics, plasma proteins, and probiotics achieved more rapid normalization of faecal consistency compared with dogs fed a standard highly digestible control diet (29). Additionally, review articles on canine acute diarrhoea management consistently recommend highly digestible diets with controlled fat and appropriate fibre content to support intestinal healing and improve nutrient absorption during episodes of gastroenteritis (30).

Due to the dehydration and possible hypovolaemia that result from gastroenteritis (1), fluid replacement therapy is a mainstay treatment of the condition. Dehydration refers to an overall decrease in total body water, while hypovolaemia refers specifically to a decrease in the effective circulating blood volume (35). Vomiting and diarrhoea cause dehydration, and where dehydration becomes severe, hypovolaemia can ensue. In this study, 72/88 (81.8%) of veterinarians administered fluids in their treatment, with most 65/72 (90.3%) employing the intravenous route of administration. The other 7/72 (9.7%) veterinarians utilised the subcutaneous (SC) route of administration. The extent of dehydration is usually assessed clinically on a scale of 5–15% (of the animal’s body weight) (20), with dehydration less than 5% being undetectable clinically. Patients that have mild to no signs of dehydration can be given SC fluids. Where a patient is not clinically dehydrated but has an ongoing history of fluid loss from vomiting and/or reduced fluid intake, the patient can be assigned a dehydration status of 5% (36). With ongoing fluid losses due to GI disease and concurrent reduced fluid intake, a patient may eventually become dehydrated or hypovolaemic, and hence, fluid therapy is indicated. Once a patient is clinically dehydrated the (IV) route of administration is preferred. The therapy should then be adjusted once the patient is stabilized to reestablish and maintain hydration over 24 h preferably. For patients receiving fluids whose dehydration status was reported, the majority (50/58, or86.2%), were clinically dehydrated, i.e., ≥5% dehydrated. Thus, the use of IV fluids in most, i.e., 46/50 (92%) of these patients, was warranted.

The major signs of gastroenteritis, vomiting and diarrhoea, usually cause isotonic dehydration (37). In this case, a replacement fluid that is isotonic to the extracellular fluid (more specifically, of a similar sodium concentration to the extracellular fluid (ECF) (37) is indicated to restore the fluid lost. Both LRS and normal saline (NS) are isotonic and can be used for this purpose, and this is most likely why LRS and 0.9% NS were the most utilised fluid. Of the veterinarians that administered fluids, 73.6 and 34.7% used LRS and 0.9%NS, respectively. While both LRS and 0.9% NS are isotonic crystalloid fluids, LRS is a balanced (buffered) isotonic crystalloid whereas 0.9% NS is not balanced. Where metabolic acidosis is present, for, e.g., in parvoviral enteritis, a buffered alkalinizing isotonic solution such as LRS is indicated for removal of H^+^ ions from the extracellular fluid (38). The metabolic acidosis in canine parvovirus primarily results from severe diarrhoea causing bicarbonate loss, and dehydration leading to reduced tissue perfusion and lactic acid buildup (39).

Five percent (5%) dextrose in 0.9% saline, a hypertonic fluid, was utilized by 16/72 (22.2%) of the veterinarians administering fluid, with 15/16 (93.8%) of these veterinarians having diagnosed parvoviral enteritis. Hypoglycaemia is a common occurrence in parvoviral enteritis (40). It is therefore highly probable that these 15 veterinarians utilizing 5% dextrose in 0.9% saline chose that fluid to treat the hypoglycaemia typically present in parvoviral enteritis. It is important to note that this 5% dextrose in 0.9% saline should not be used in a dehydrated patient as it causes diuresis (and therefore further fluid loss) before sufficient plasma volume has been obtained (41). The patient must first be adequately rehydrated as can be accomplished with an isotonic crystalloid, and then a dextrose-containing solution can be administered to address hypoglycaemia. As 11 of the aforementioned 15 (73.3%) veterinarians utilised both LRS and 5% dextrose in 0.9% saline, it is probable that they would have first administered the LRS to rehydrate their parvovirus patient, before following up with the 5% dextrose in 0.9% saline to address the hypoglycaemia that may have been present.

In this study, it was observed that 93.2% of veterinarians utilized broad-spectrum antimicrobials in the treatment of their gastroenteritis case. The route of administration was not captured in the survey utilized in this study, thus such information could not be discussed. The majority of veterinarians that diagnosed bacterial enteritis utilized the presenting clinical signs (vomiting, fever, haemorrhagic and nonhaemorrhagic diarrhoea, dehydration, anorexia and lethargy) to aid their diagnosis, while a few (3/15) did a complete blood count (CBC), most likely for identification of an increase in the white blood cell count. Since most veterinarians used broad-spectrum antibiotics regardless of the cause of gastroenteritis (bacterial, parvoviral, endoparasitic), it is possible that the reason for antibiotic usage was to prevent secondary systemic bacterial infection from bacterial translocation due to the compromised intestinal mucosa, and the puppies’ immature immune system (3). It should be noted that these findings reflect reported clinical practices among the surveyed veterinarians and do not necessarily represent guideline-aligned or optimal standards of care.

The results from this study indicate that metronidazole was the most frequently used antibiotic (51.2%) for treating gastroenteritis in puppies up to 1 year of age. The preference for metronidazole in Trinidad and Tobago and Barbados could have been due to its anti-inflammatory properties and action against anaerobic bacteria, as well its antiprotozoal activity (42). However, research suggests that metronidazole may disrupt gut microbiota, leading to long-term consequences on gastrointestinal health as well as cause nephrotoxicity (43). The second most used antibiotic was Amoxicillin (35.4%), a broad-spectrum beta lactam antibiotic which targets gram negative, gram positive and anaerobic organisms. The third most used antibiotic was Trimethoprim-Sulfamethoxazole (24.4%). The spectrum of sulfadimethoxine is broad and includes gram negative, gram positive as well as anaerobic organisms (44).

The predominance of metronidazole use in the present study is similar to findings reported in previous studies evaluating treatment practices for acute canine diarrhoea, where metronidazole remains one of the most frequently prescribed antimicrobials despite increasing concerns regarding antimicrobial stewardship and gastrointestinal dysbiosis (45). However, the frequent use of antibiotics in uncomplicated gastroenteritis cases may reflect a broader trend reported internationally in small animal practice, where antimicrobials are often prescribed empirically even when evidence supporting clinical benefit is limited (46).

The overuse of antibiotics in veterinary medicine is a growing concern. Antibiotics are often prescribed as a first-choice therapy in over 50% of dogs presenting with acute diarrhoea in the UK. However, this practice may contribute to the development of antimicrobial resistance and can disrupt the gut microbiota, potentially leading to adverse effects (47). A study done in 2020 by the Royal Veterinary College (RVC) analyzed data from 894 dogs diagnosed with uncomplicated diarrhoea. The findings revealed that 88.3% of dogs prescribed antibiotics showed clinical resolution, compared to 87.9% of dogs that did not receive antibiotics (a negligible difference of 0.4%). This indicates that antibiotics did not provide a significant benefit in treating uncomplicated diarrhoea (48). Furthermore, guidelines released recently by the European Network for Optimization of Veterinary Antimicrobial Therapy (ENOVAT) strongly advise that antimicrobial use should be reserved only for the most severe cases of acute diarrhoea, for instance, in cases of haemorrhagic or non-haemorrhagic diarrhoea with severe disease, i.e., cases with impaired general condition, hypovolemia, and signs of systemic disease despite adequate fluid therapy (49). Treatment response should be assessed daily if hospitalized, and antibiotic therapy should not extend beyond resolution of clinical signs which is typically within 3–7 days for the majority of dogs. Another study showed that the use of antibiotics in dogs with AHDS is unnecessary unless sepsis is present (8). However, with respect to Parvoviral enteritis, a 2020 update on the disease (40), stated that CPV patients are “at high risk for bacterial translocation” because of the intestinal villous collapse and decreased immune function caused by the virus. The article also stated that broad-spectrum antibiotics are recommended for all CPV patients (i.e., septic or not). Hence, there is merit for veterinarians’ use of antibiotics when treating a patient with parvoviral enteritis. Nevertheless, in cases where antibiotics are not necessarily indicated, it should be noted that alternative treatments such as probiotics have been viewed as a safer option for restoring gut health (50).

Twenty-seven veterinarians (27/88, i.e., 30.7%) utilized analgesics in treatment of their gastroenteritis case. In Figure 6, it was observed that meloxicam was the analgesic most frequently used by veterinarians (18/27, i.e., 66.7%). It is likely that this drug was used as it is a preferential COX-2 inhibitor thus limiting COX 1 activity which is responsible for gastric mucosal integrity/protection, renal blood flow and platelet aggregation (51). Additionally, the current approach emphasizes administering meloxicam with food to minimize potential gastrointestinal side effects and allow safe administration (52). Thus, by limiting COX 1 activity and giving it with food, the animal’s gastric mucosa, already compromised due to gastroenteritis, is at lower risk of further damage. Still, administration of meloxicam in cases of gastroenteritis should be approached with caution. The use of non-steroidal anti-inflammatory drugs (NSAIDs) in patients with gastrointestinal disease remains controversial due to their potential to impair gastric mucosal protection through inhibition of prostaglandin synthesis. This may increase the risk of gastrointestinal irritation, ulceration, or delayed mucosal healing, particularly in patients with vomiting, diarrhoea, or compromised perfusion. Clinicians must carefully balance the benefits of analgesia against these potential risks, particularly when the patient’s hydration status is uncertain (53).

Tramadol was the second most commonly used analgesic by veterinarians (5/27, i.e., 18.5%) in the treatment of their gastroenteritis case. The majority (4/5, i.e., 80%) utilized it as the sole analgesic, however, tramadol should preferably be used as a component of multimodal analgesic therapy. O-desmethyltramadol (M1) is the main active metabolite responsible for the analgesic effects of tramadol. However, in dogs, the inactive N-desmethyltramadol metabolite is produced at a faster rate, resulting in lower levels of the active M1 metabolite. As a result of the reduced formation of the active metabolite, tramadol’s pain-relieving effects in dogs are variable making the sole use of tramadol inadequate for pain management (54). Gastroenteritis, particularly canine parvoviral enteritis, can cause significant abdominal pain and discomfort. Additionally, tramadol provides pain relief without causing adverse effects associated with NSAIDs such as GI ulcer formation, gastritis and further compromise the mucosal integrity (55). However, as aforementioned, it is not adequate for analgesia in dogs when used as the sole agent (54).

Hyoscine butylbromide was the least used (7.4%) as it is an antispasmodic and not a true analgesic. It can be used to potentially alleviate abdominal cramping by reducing smooth muscle contractions associated with gastroenteritis thereby warranting its use (56). It can also reduce spasms in the GIT, which may provide symptomatic relief in acute gastroenteritis (56). However, if the gastroenteritis has an underlying infectious cause, it can only offer symptomatic relief and not address the underlying infection and its associated effects such as fever thereby warranting its sparse use (57).

Other studies evaluating analgesic protocols in canine gastroenteritis and parvoviral enteritis have generally emphasized the use of opioid-based analgesia rather than NSAIDs, particularly in hospitalized or critically ill puppies (40). Buprenorphine, butorphanol, fentanyl and methadone are commonly recommended because they provide effective visceral analgesia while minimizing the risk of gastrointestinal ulceration and renal hypoperfusion associated with NSAID administration in dehydrated patients. One study demonstrated that meloxicam administration may affect gastrointestinal permeability in dogs, reinforcing concerns regarding NSAID use in patients with gastrointestinal disease (58). In contrast, opioid analgesics such as butorphanol and buprenorphine are more frequently recommended in severe Canine Parvoviral Enteritis (CPE) cases due to their analgesic efficacy and reduced gastrointestinal adverse effects compared with NSAIDs (59). Overall, current literature supports individualized and multimodal analgesic strategies in dogs with severe gastroenteritis to improve patient comfort while limiting adverse gastrointestinal effects.

Antiemetic administration was done by 75/88 (85%) veterinarians in order to decrease ongoing fluid and electrolyte losses (2) since 39/88 (44%) and 59/88 (67%) veterinarians reported vomiting and dehydration, respectively. Antiemetic therapy also allows for earlier reintroduction of enteral nutrition (2). This is consistent with current recommendations for management of canine gastroenteritis and parvoviral enteritis, where controlling emesis is important for reducing ongoing fluid loss, improving patient comfort, and facilitating enteral nutrition (40). In this study, metoclopramide was the most frequently used antiemetic (55.6%) and while maropitant is the only licensed antiemetic for use in dogs, it was the second most frequently used antiemetic (41.7%).

Of the 64 veterinarians that diagnosed parvovirus, 59 (92.2%) administered antiemetics, with 33/59 (55.9%) opting to use Metoclopramide. It is possible that veterinarians preferred to administer metoclopramide due to its additional prokinetic effect as parvovirus causes gastric hypomotility (60). Additionally, clients may have opted for the use of metoclopramide over maropitant due to its lower price point and little difference in clinical effect between the two (61). However, excessive prokinetic stimulation with metoclopramide may theoretically increase the risk of gastrointestinal complications such as intussusception, whereas maropitant, in addition to its antiemetic effects, has been shown to possess visceral analgesic properties (62, 63).

Some veterinarians also prefer to administer metoclopramide as it can be given as a continuous rate infusion (CRI) in the IV fluids (64). It must also be noted that maropitant should not be used in puppies under 8 weeks of age as it could result in a hypocellular bone marrow which could have detrimental effects if the puppy already has a haemorrhagic diarrhoea (65). Maropitant should also be used cautiously when giving highly protein bound drugs such as NSAIDs and sulfa class antibiotics (66). While metoclopramide was the most used antiemetic by veterinarians with 0–5, 6–10, 21–25 and 36–40 years of activity, maropitant was the mode for veterinarians with 11–15 years of activity. Maropitant (Cerenia®) was released in 2007 for treatment of canine emesis. This would have been in the early career of veterinarians with 11–15 years of activity, and it is plausible that they would have begun using maropitant as it was a newly available FDA approved drug.

Probiotics are defined as live microorganisms which when administered in adequate amounts confer a health benefit (67). Probiotics add to the population of beneficial gut microbes. It was observed that 36/88 (40.9%) veterinarians utilized probiotics, of which Fortiflora® was the most commonly used, with 23/36 (63.9%) veterinarians using it. This suggests that Fortiflora® is the preferred probiotic for managing gastroenteritis in puppies, likely due to its proven efficacy in restoring gut microbiota balance (9). The use of probiotics in the present study is consistent with increasing utilization of microbiome-targeted therapies in small animal veterinary medicine for the management of acute diarrhoea (68).

According to the U.S. Food and Drug Administration (FDA), the term “nutraceutical” is not officially recognized under U.S. law. Instead, nutraceuticals typically fall under the categories of dietary supplements, food additives, or functional foods. The FDA defines dietary supplements as products intended to supplement the diet that contain vitamins, minerals, herbs, amino acids, or other substances meant to enhance health. Unlike pharmaceuticals, they are not intended to diagnose, treat, cure, or prevent diseases (69). Figure 9 highlighted that vitamins were the most commonly used nutraceutical (85.3%) in the treatment of gastroenteritis in puppies. The frequent use of vitamins suggests that veterinarians prioritize supportive care and boosting the immune system when managing gastroenteritis.

The frequent use of vitamins and nutraceuticals in the present study is consistent with supportive treatment approaches commonly utilized in small animal veterinary medicine for managing gastroenteritis, particularly in puppies with reduced appetite, dehydration and impaired nutrient absorption. Previous veterinary literature has emphasized the importance of supportive care in gastrointestinal disease management, especially in cases of parvoviral enteritis where intestinal damage, vomiting and diarrhoea may contribute to nutritional deficiencies and delayed recovery (45). The high prevalence of parvoviral enteritis observed in the present study may therefore explain the frequent incorporation of vitamin supplementation as part of the therapeutic protocol.

In this study, 58/88 (65.9%) veterinarians administered vitamins with multivitamins being the most frequently used (24/58 or 41.4%). Most multivitamins are formulated with B vitamins, vitamin A, C, D and E, calcium, phosphorus and Omega 3 fatty acids (70). The second most frequently used vitamin supplement was Vitamin B 12. This is crucial for red blood cell production (71), which is necessary in cases of haemorrhagic diarrhoea, seen in parvovirus and AHDS. Canine parvovirus enteritis also leads to the destruction of enterocytes, resulting in malabsorption. This damage impairs the absorption of vitamin B12, which is essential for DNA synthesis and the regeneration of enterocytes. Consequently, parenteral administration of cobalamin is often necessary in affected dogs. Studies have shown that dogs with parvoviral enteritis frequently exhibit hypocobalaminaemia (low serum B12 levels), and supplementation should be considered as part of their therapeutic management (72). The frequent use of multivitamins and vitamin B12 in this study suggests that veterinarians commonly incorporate supportive therapies into management of canine gastroenteritis, particularly in debilitated or anorexic patients. Similar supportive approaches have been described in canine parvoviral enteritis, where gastrointestinal damage and malabsorption may contribute to hypocobalaminaemia and delayed enterocyte recovery (73). (Additionally, Vitamin B12 promotes cell growth and replication, along with a boost in appetite, which can help hasten recovery from gastroenteritis (74). Vitamin C was administered by a small percentage (3.4% i.e., 3/88) of veterinarians. Ascorbic acid (vitamin C) is not an essential dietary vitamin in dogs, as it is synthesized endogenously in the liver. Its use may therefore represent a supportive or adjunctive therapy rather than correction of deficiency. Clinically, it may be administered for its proposed antioxidant effects and potential role in supporting immune function during inflammation. However, evidence for its benefit in canine gastroenteritis is limited, and the rationale for its use may be empirical, likely reflecting clinicians’ preferences (75). While there is no published evidence suggesting the role of vitamins in the treatment of gastroenteritis in dogs, one can deduce that it was administered to support the animal’s immune system, stimulate appetite, and supplement vitamins that cannot be absorbed in the gastrointestinal tract due to the gastroenteritis (76).

Twenty-five (25/88 or 28.54%) veterinarians used appetite stimulants of which cyproheptadine was the most used (76%) to treat their gastroenteritis case. In cases of gastroenteritis, puppies experience reduced appetite due to nausea and GI discomfort. Thus, cyproheptadine which is an antihistamine can be used to combat resultant anorexia due to its additional anti-serotonergic effects which promotes increased food intake (77). Due to its mechanism of action, it can allow the animal to regain energy levels and support the nutritional needs essential for its recovery which may have warranted its primary use. Similar nutritional support strategies have been recommended in veterinary gastrointestinal management, where improving caloric intake is considered important for recovery in anorexic patients (78). (This supports the relatively frequent use of cyproheptadine observed in this study, as veterinarians may have prioritized restoration of appetite and nutritional intake in debilitated gastroenteritis cases. Mirtazapine was the second most commonly used appetite stimulant (12%). It is a tetracyclic antidepressant that antagonizes serotonin (5-HT2 and 5-HT3) and histamine (H1) receptors (79) thereby reducing nausea and enhancing appetite. Most studies and clinical experiences focus on cats, leaving a gap in knowledge regarding its effects on dogs (80). This may be why it was not the primary appetite stimulant used. One veterinarian utilized capromorelin, the first ghrelin receptor agonist approved in the US for treatment of anorexia in both dogs and cats.

The total study population comprised of veterinarians from Trinidad and Tobago and Barbados. One limitation of this study is that responses from both countries were analyzed together, masking possible variation between the two countries’ approach to treatment. Additionally, because there were more responses from Trinidad (58/88) than Barbados (30/88), the combined results may be more representative of clinical practice in Trinidad and Tobago. Still, the markedly low response rate for Trinidad and Tobago (33.8%, i.e., 58 out of the sample size of 173) precludes generalization of the combined results to the veterinary population there. As the response rate for Barbados was significantly higher (30/42, i.e., 71.4%), additional analysis was done on the data obtained from veterinarians in this country solely. This allowed for creation of an algorithm representative of veterinarians’ approach to treatment in Barbados, specifically.

The algorithm presented (Figure 10) reflects the most reported practices among respondents rather than established clinical guidelines. For example, the reported cases of endoparasitism were treated with fluid therapy, reflecting supportive care priorities but not addressing the routine use of anthelmintic treatment in such cases. The authors suspect that some veterinarians elected not to administer dewormers based on a reported history of deworming prior to presentation. However, the timing and adequacy of prior deworming were not ascertained as this information was not captured in the questionnaire. Similarly, the inclusion of antibiotics in the management of parvoviral enteritis may appear inconsistent with its viral aetiology. However, antibiotic therapy is often employed in these patients due to the high risk of secondary bacterial translocation associated with intestinal mucosal damage and immunosuppression. This rationale was not explicitly captured in the survey and may not be immediately evident from the algorithm. Therefore, the algorithm should be interpreted solely as a representation of reported clinical practices rather than a prescriptive treatment protocol.

Several studies investigating canine gastroenteritis have reported treatment approaches comparable to those illustrated in Figure 10, with management primarily focused on supportive and symptomatic care. Fluid therapy is described as the cornerstone of treatment since vomiting and diarrhoea usually result in dehydration, electrolyte disturbances and acid–base imbalances (81). Antiemetics, including metoclopramide and maropitant, are also commonly incorporated to control vomiting and improve patient comfort and feed tolerance (81, 82). In studies evaluating canine parvoviral enteritis, supportive care commonly include intravenous fluid therapy, antiemetics, antimicrobial therapy and nutritional support as part of a well-rounded approach to patient stabilization (81, 82).

In this study, there was heterogeneity in treatment protocols among vets with varying years of activity in the field. This may be due to continuous updating of the available body of scientific information, which would have influenced the teaching and learning materials at the point in time in which the veterinarian attended school. It can be speculated that not all veterinarians would be up to date with current research publications and new treatments due to the overwhelming volume of new developments (83), as well as the busy schedules and long hours characteristic of veterinary clinical practice. Thus, it is plausible that some may still rely primarily on the knowledge acquired during their veterinary schooling. The regulatory framework for the veterinary profession is an important factor to consider. In many European countries, continuing professional development (CPD) is a requirement for maintaining veterinary licensure through regulatory bodies such as the Royal College of Veterinary Surgeons (RCVS). The RCVS mandates a specified amount of annual CPD for all registered veterinarians (84). Veterinarians’ adherence to this requirement should aid in keeping clinical practice aligned with current evidence-based standards through regular updating of knowledge. This may help reduce differences in clinical decision-making among veterinarians with different levels of experience.

In contrast, CPD requirements are not present in all Caribbean countries. In Jamaica, registered veterinarians are required to complete a certain number of continuing education credits annually in order to maintain their licensure to practice (85). In other Caribbean countries such as Barbados and Trinidad and Tobago, there are no similar requirements for maintaining licensure. While some veterinarians may engage in self-directed CPD, it is quite understandable that it may not be as consistent as if concrete requirements and guidelines necessitating CPD were in place. As a result, some practitioners may depend more on the knowledge gained during their initial training, along with personal clinical experience. This difference in regulatory oversight may help explain the variation in treatment approaches observed in this study, reflecting not only individual preferences but also differences in professional accountability to continuing education.

Given the rapid evolution of veterinary medicine, continuous updating of clinical knowledge is essential to ensure that treatment protocols remain effective, safe, and evidence-based. New research may change recommendations regarding various aspects of gastroenteritis management in puppies. Ongoing professional development helps clinicians adapt to emerging pathogens, regional disease trends, and advances in therapeutics, ultimately improving patient outcomes and standards of care. In resource-variable settings such as Trinidad and Tobago and Barbados, staying current also supports more practical and locally relevant clinical decision-making.

## 5 Conclusion

This study explored the diagnostic and treatment options utilized by veterinarians in Trinidad and Tobago and Barbados for the treatment of gastroenteritis in puppies. Intravenous fluid therapy, antibiotics, and antiemetics were utilized by most participating veterinarians. Additionally, the administration of probiotics, appetite stimulants, and nutraceuticals by a significant proportion of respondents indicated an increasing recognition of supportive therapies in gastrointestinal recovery.

## Data Availability

The original contributions presented in the study are included in the article/Supplementary material, further inquiries can be directed to the corresponding author/s.
