# Supplementary material for: Managing gastroenteritis in puppies: veterinary practices in Trinidad and Tobago and Barbados
Source: Front Vet Sci. 2026 Sep 29;13:1813429. doi: 10.3389/fvets.2026.1813429 (PMC13659807; doi:10.3389/fvets.2026.1813429)
Supplement: Supplementary file 1 [file Data_Sheet_1.pdf]

The Approach of Veterinarians in Trinidad & Tobago and Barbados to Treating Gastroenteritis in Puppies

* Indicates required question

1. Do you agree to participate in this study? *

   Mark only one oval.

   ( ) Yes

Client consent

2. Has client consent been obtained to provide information about their pet that will be collected when answering the questions of this survey? *

   Mark only one oval.

   ( ) Yes

   ( ) No

Background

Please indicate your clinic area. If you are a mobile veterinarian, please tick the option indicating such.

3. Clinic Area (TRINIDAD & TOABGO)

Mark only one oval.

- ( ) Caroni
- ( ) Mayaro
- ( ) St. Andrew (Trinidad)
- ( ) St. David
- ( ) St. George (Trinidad)
- ( ) St. Patrick (Trinidad)
- ( ) Victoria
- ( ) St. Andrew (Tobago)
- ( ) St. David (Tobago)
- ( ) St. George (Tobago)
- ( ) St. John (Tobago)
- ( ) St. Mary (Tobago)
- ( ) St. Patrick (Tobago)
- ( ) St. Paul (Tobago)
- ( ) N/A (Mobile Veterinarian)
- ( ) Other: ____

4. Clinic Area (BARBADOS)

Mark only one oval.

- St Lucy
- St Peter
- St Andrew
- St James
- St Joseph
- St George
- St Thomas
- St John
- St Michael
- St Philip
- Christ Church
- N/A (Mobile Veterinarian)

5. Years of veterinary experience *(to the nearest whole number)* and type of practice (*eg small, mixed*) *

____

Signalment

6. Breed of Dog *

Mark only one oval.

- Akita
- Cocker Spaniel
- Daschund
- German Shepherd
- Pitbull
- Rottweiler
- Siberian Husky
- Mastiff
- Maltese
- Miniature Pinscher
- Miniature Poodle
- Toy Poodle
- Yorkshire Terrier
- English Bulldog
- French Bulldog
- Belgian Mallinois
- Mixed Breed
- Other: ____

7. Sex *

Mark only one oval.

- ⬭ Male
- ⬭ Female

8. Weight (to the nearest whole number in either kg or lbs) *

____

9. Age *

Mark only one oval.

- ⬭ < 1 month
- ⬭ 1-2 months
- ⬭ 3-4 months
- ⬭ 5-6 months
- ⬭ 7-8 months
- ⬭ 9-10 months
- ⬭ 11-12 months

History

Clinical Signs and Deworming Status

10. Please select the clinical signs displayed by the patient *

Check all that apply.

- [ ] Vomiting
- [ ] Hemorrhagic diarrhea with foul odor
- [ ] Hemorrhagic diarrhea without odor
- [ ] Diarrhea without blood
- [ ] Fever
- [ ] Depression
- [ ] Anorexia
- [ ] Abdominal pain

11. Please state the dehydration status of the patient (eg 5%). *
(Write n/a if not known)

____

12. Before admission, how long had patient been *

Check all that apply.

	1 d	2 d	3 d	4 d	5 d	6 d	7 d	>1 week	N/.
**Vomiting**	[ ]	[ ]	[ ]	[ ]	[ ]	[ ]	[ ]	[ ]	[ ]
**Diarrhoea**	[ ]	[ ]	[ ]	[ ]	[ ]	[ ]	[ ]	[ ]	[ ]
**Anorexic**	[ ]	[ ]	[ ]	[ ]	[ ]	[ ]	[ ]	[ ]	[ ]

13. Was a fecal smear done? *

Mark only one oval.

- ( ) Yes
- ( ) No

14. If yes, what was found?

____

15. Had the patient been dewormed (prior to the visit with gastroenteritis)? *

Mark only one oval.

( ) Yes *Skip to question 16*

( ) No *Skip to question 17*

Skip to question 16

Deworming

16. Which dewormer had been used on the patient's last deworming? *

____

Vaccination Status

17. Had the patient been vaccinated? *

Mark only one oval.

( ) Fully vaccinated *Skip to question 18*

( ) Partially vaccinated *Skip to question 18*

( ) Not vaccinated *Skip to question 19*

( ) Do not know *Skip to question 19*

Vaccinations

18. Which vaccines had the patient received? *

Check all that apply.

- [ ] Canine Leptospirosis
- [ ] Canine Parvovirus
- [ ] Canine Rabies
- [ ] Kennel Cough
- [ ] Nobivac DHPPi (Canine Distemper, Adenovirus, Parvovirus, Parainfluenza) Vaccine
- [ ] Nobivac DAPPv (Distemper- Adenovirus Type 2 - Parainfluenza - Parvovirus) Vaccine
- [ ] Nobivac Lepto4 (Leptospira canicola, L. icterohaemorrhagiae, L. pomona, L. grippotyphosa)
- [ ] Other: ______________

Diagnosis

19. Which of the following did you identify as the cause of gastroenteritis in the patient?

Check all that apply.

- [ ] Bacterial infection
- [ ] Endoparastism
- [ ] Parvovirus
- [ ] Other viral infection
- [ ] Foreign body
- [ ] Unknown cause
- [ ] Other: ______________

20. Which of the following clinical signs influenced your working diagnosis? *

Check all that apply.

- [ ] Dehydration
- [ ] Vomiting
- [ ] Diarrhea (without blood)
- [ ] Hemorrhagic diarrhea
- [ ] Foul odour with diarrhea
- [ ] Fever
- [ ] Loss of appetite
- [ ] Lethargy
- [ ] Depression
- [ ] Other: ____________________

21. Which of the following diagnostic tests further assisted in arrival at the definitive diagnosis?

Check all that apply.

- [ ] X-ray
- [ ] Ultrasonography
- [ ] Palpation
- [ ] Parvo ELISA Test
- [ ] 4Dx PLUS SNAP Test
- [ ] SNAP Giardia Test
- [ ] Faecal flotation
- [ ] Faecal sedimentation
- [ ] N/A
- [ ] Other: ____________________

Treatment

22. Was the patient hospitalized (kept for >1 day)? *

Mark only one oval.

( ) Yes

( ) No

23. Were fluids administered? *

Mark only one oval.

- ( ) Yes *Skip to question 24*
- ( ) No *Skip to question 26*

Fluids

24. Which fluids were administered, via what route?

Check all that apply

Mark only one oval per row.

	IV	SC
**0.9% Saline**	( )	( )
**Lactated Ringer's solution**	( )	( )
**2.5% dextrose in 0.45% saline**	( )	( )
**5% dextrose saline**	( )	( )
**Plasma**	( )	( )
**Dextrans**	( )	( )
**Hydroxyethyl starch**	( )	( )
**Lyophilized canine albumin**	( )	( )
**N/A**	( )	( )

25. Were any fluids not mentioned above administered? If yes, please state which. *

____

26. Were analgesics administered? *

Mark only one oval.

- ( ) Yes *Skip to question 27*
- ( ) No *Skip to question 28*

Analgesia

27. Which analgesics were administered? *

Check all that apply.

- [ ] Carprofen
- [ ] Deracoxib
- [ ] Fentanyl
- [ ] Gabapentin
- [ ] Grapiprant
- [ ] 2% lidocaine
- [ ] Morphine
- [ ] Tramadol
- [ ] Meloxicam
- [ ] Other: ________________________________

28. Were antibiotics administered? *

Mark only one oval.

- ( ) Yes *Skip to question 29*
- ( ) No *Skip to question 30*

Antibiotics

29. Which antibiotics were administered? *

Check all that apply.

- [ ] Amoxicillin
- [ ] Ampicillin
- [ ] Enrofloxacin
- [ ] Metronidazole
- [ ] Other: ______________________

30. Were antiemetics administered? *

Mark only one oval.

- ( ) Yes *Skip to question 31*
- ( ) No *Skip to question 32*

Antiemetics

31. Which antiemetics were administered? *

Check all that apply.

- [ ] Dolasetron
- [ ] Maropitant
- [ ] Metoclopramide
- [ ] Ondansetron
- [ ] Phenothiazines
- [ ] Other: ______________________

32. Were probiotics administered? *

Mark only one oval.

- ( ) Yes *Skip to question 33*
- ( ) No *Skip to question 34*

Probiotics

33. Which probiotics were administered? *

____

34. Were vitamins administered? *

Mark only one oval.

- ( ) Yes *Skip to question 35*
- ( ) No *Skip to question 36*

Vitamins

35. Which vitamins were administered? *

____

36. Were appetite stimulants administered? *

Mark only one oval.

- ( ) Yes *Skip to question 37*
- ( ) No *Skip to question 38*

Appetite Stimulants

37. Which appetite stimulants were administered? *

Check all that apply.

- [ ] Capromorelin
- [ ] Cyproheptadine
- [ ] Mirtazapine
- [ ] Other: _______________

38. Were iron supplements administered? *

Mark only one oval.

- ( ) Yes *Skip to question 39*
- ( ) No *Skip to question 40*

Iron Supplementation

39. Which iron supplement(s) were administered? *

____

40. Were nutraceuticals administered? *

Mark only one oval.

- ( ) Yes *Skip to question 41*
- ( ) No *Skip to question 42*

Nutraceuticals

41. Which nutraceuticals were administered? *

____

42. Were any other treatments not mentioned above administered? *

Mark only one oval.

- ( ) Yes *Skip to question 43*
- ( ) No *Skip to question 44*

43. Please state the other treatment(s) administered *

____

Outcome

44. Did the patient survive? *

Mark only one oval.

- Yes
- No
- Information not available

This content is neither created nor endorsed by Google.

Google Forms
