# Supplementary material for: Managing gastroenteritis in puppies: veterinary practices in Trinidad and Tobago and Barbados
Source: Front Vet Sci. 2026 Sep 29;13:1813429. doi: 10.3389/fvets.2026.1813429 (PMC13659807; doi:10.3389/fvets.2026.1813429)
Supplement: Supplementary file 2 [file Supplementary_file_1.docx]

 Supplemental Figure 1. Proportions of Surveyed Veterinarians that Used Fluids

Supplemental Figure 2. Vitamins used by Surveyed Veterinarians in T&T and Barbados

Supplemental Figure 3. Probiotics used by surveyed veterinarians in T&T and Barbados

Supplemental Figure 4. Percentage of Surveyed Veterinarians with Various Years of Activity

Supplemental Figure 5a. Fluids used with varying years of activity (0-20 years)

Supplemental Figure 5b. Fluids used with varying years of activity (21-45 years)

Supplemental Figure 6a. Antibiotics used with varying years of activity (0-10 years)

Supplemental Figure 6b. Antibiotics used with varying years of activity (11-20 years)

Supplemental Figure 6c. Antibiotics used with varying years of activity (21-30 years)

Supplemental Figure 6d. Antibiotics used with varying years of activity (36-45 years)

Supplemental Figure 7. Analgesics used with varying years of activity (0-20 years)

Supplemental Figure 8a. Antiemetics used with varying years of activity (0-15 years)

Supplemental Figure 8b. Antiemetics used with varying years of activity (16-40 years)

Supplemental Figure 9a. Probiotics used with varying years of activity (0-15 years)

 Supplemental Figure 9b. Probiotics used with varying years of activity (16-25 years) Supplemental Figure 10a. Nutraceuticals used with varying years of activity (0-15 years)

Supplemental Figure 10b. Nutraceuticals used with varying years of activity (16-25 years)
